# Characterization of spurious-electron signals in the double-phase argon TPC of the DarkSide-50 experiment

**DOI:** 10.1140/epjc/s10052-026-16193-y

**Published:** 2026-09-23

**Authors:** DarkSide-50 Collaboration, P. Agnes, I. F. Albuquerque, T. Alexander, A. K. Alton, M. Ave, H. O. Back, G. Batignani, E. Berzin, K. Biery, V. Bocci, W. M. Bonivento, B. Bottino, S. Bussino, M. Cadeddu, M. Cadoni, F. Calaprice, A. Caminata, M. D. Campos, N. Canci, M. Caravati, N. Cargioli, M. Cariello, M. Carlini, V. Cataudella, P. Cavalcante, S. Cavuoti, S. Chashin, A. Chepurnov, C. Cicalò, G. Covone, D. D’Angelo, S. Davini, A. De Candia, S. De Cecco, G. De Filippis, G. De Rosa, A. V. Derbin, A. Devoto, M. D’Incecco, C. Dionisi, F. Dordei, M. Downing, D. D’Urso, M. Fairbairn, G. Fiorillo, D. Franco, F. Gabriele, C. Galbiati, C. Ghiano, C. Giganti, G. K. Giovanetti, A. M. Goretti, G. Grilli di Cortona, A. Grobov, M. Gromov, M. Guam, M. Gulino, B. R. Hackett, K. Herner, T. Hessel, B. Hosseini, F. Hubaut, T. Hugues, E. V. Hungerford, A. Ianni, V. Ippolito, K. Keeter, C. L. Kendziora, M. Kimura, I. Kochanek, D. Korablev, G. Korga, A. Kubankin, M. Kuss, M. Kuźniak, M. La Commara, M. Lai, X. Li, M. Lissia, G. Longo, O. Lychagina, I. N. Machulin, L. P. Mapelli, S. M. Mari, J. Maricic, A. Messina, R. Milincic, J. Monroe, M. Morrocchi, V. N. Muratova, P. Musico, A. O. Nozdrina, A. Oleinik, F. Ortica, L. Pagani, M. Pallavicini, L. Pandola, E. Pantic, E. Paoloni, K. Pelczar, N. Pelliccia, S. Piacentini, A. Pocar, M. Poehlmann, S. Pordes, S. S. Poudel, P. Pralavorio, D. Price, F. Ragusa, M. Razeti, A. Razeto, A. L. Renshaw, M. Rescigno, J. Rode, A. Romani, D. Sablone, O. Samoylov, E. Sandford, W. Sands, S. Sanfilippo, C. Savarese, B. Schlitzer, D. A. Semenov, A. Sheshukov, M. D. Skorokhvatov, O. Smirnov, A. Sotnikov, R. Stefanizzi, S. Stracka, C. Sunny, Y. Suvorov, R. Tartaglia, G. Testera, A. Tonazzo, E. V. Unzhakov, A. Vishneva, R. B. Vogelaar, M. Wada, H. Wang, Y. Wang, S. Westerdale, M. M. Wojcik, X. Xiao, C. Yang, G. Zuzel

**Affiliations:** 1 Gran Sasso Science Institute, 67100 L’Aquila, Italy; 2 INFN Laboratori Nazionali del Gran Sasso, 67100 Assergi, AQ Italy; 3 Instituto de Física, Universidade de São Paulo, São Paulo, 05508-090 Brazil; 4 Pacific Northwest National Laboratory, Richland, WA 99352 USA; 5 Physics Department, Augustana University, Sioux Falls, SD 57197 USA; 6 Physics Department, Università degli Studi di Pisa, 56127 Pisa, Italy; 7 INFN Pisa, 56127 Pisa, Italy; 8 Fermi National Accelerator Laboratory, Batavia, IL 60510 USA; 9 INFN Sezione di Roma, 00185 Rome, Italy; 10 INFN Cagliari, 09042 Cagliari, Italy; 11 Physics Department, Università degli Studi di Genova, 16146 Genoa, Italy; 12 INFN Genova, 16146 Genoa, Italy; 13 Mathematics and Physics Department, Università degli Studi Roma Tre, 00146 Rome, Italy; 14 INFN Roma Tre, 00146 Rome, Italy; 15 Physics Department, Università degli Studi di Cagliari, 09042 Cagliari, Italy; 16 Physics Department, Princeton University, Princeton, NJ 08544 USA; 17 Physics, Kings College London, Strand, London, WC2R 2LS UK; 18 Virginia Tech, Blacksburg, VA 24061 USA; 19 Skobeltsyn Institute of Nuclear Physics, Lomonosov Moscow State University, Moscow, 119234 Russia; 20 Physics Department, Università degli Studi di Milano, 20133 Milan, Italy; 21 INFN Milano, 20133 Milan, Italy; 22 Physics Department, Sapienza Università di Roma, 00185 Rome, Italy; 23 Saint Petersburg Nuclear Physics Institute, Gatchina, 188350 Russia; 24 Amherst Center for Fundamental Interactions and Physics Department, University of Massachusetts, Amherst, MA 01003 USA; 25 Physics Department, Università degli Studi “Federico II” di Napoli, 80126 Naples, Italy; 26 INFN Napoli, 80126 Naples, Italy; 27 APC, Université Paris Diderot, CNRS/IN2P3, CEA/Irfu, Obs de Paris, USPC, 75205 Paris, France; 28 LPNHE, CNRS/IN2P3, Sorbonne Université, Université Paris Diderot, 75252 Paris, France; 29 INFN Laboratori Nazionali di Frascati, 00044 Frascati, Italy; 30 National Research Centre Kurchatov Institute, Moscow, 123182 Russia; 31 Institute of High Energy Physics, Beijing, 100049 China; 32 Engineering and Architecture Faculty, Università di Enna Kore, 94100 Enna, Italy; 33 INFN Laboratori Nazionali del Sud, 95123 Catania, Italy; 34 Centre de Physique des Particules de Marseille, Aix Marseille Univ, CNRS/IN2P3, CPPM, Marseille, France; 35 Department of Physics, University of Houston, Houston, TX 77204 USA; 36 School of Natural Sciences, Black Hills State University, Spearfish, SD 57799 USA; 37 AstroCeNT, Nicolaus Copernicus Astronomical Center of the Polish Academy of Sciences, 00-614 Warsaw, Poland; 38 Joint Institute for Nuclear Research, Dubna, 141980 Russia; 39 Radiation Physics Laboratory, Belgorod National Research University, Belgorod, 308007 Russia; 40 Department of Physics and Astronomy, University of California, Riverside, CA 92507 USA; 41 National Research Nuclear University MEPhI, Moscow, 115409 Russia; 42 Physics and Astronomy Department, University of California, Los Angeles, CA 90095 USA; 43 Department of Physics and Astronomy, University of Hawai’i, Honolulu, HI 96822 USA; 44 University of Oxford, Oxford, OX1 2JD UK; 45 Chemistry, Biology and Biotechnology Department, Università degli Studi di Perugia, 06123 Perugia, Italy; 46 INFN Perugia, 06123 Perugia, Italy; 47 Department of Physics, University of California, Davis, CA 95616 USA; 48 M. Smoluchowski Institute of Physics, Jagiellonian University, 30-348 Kraków, Poland; 49 The University of Manchester, Manchester, M13 9PL UK; 50 Center for Experimental Nuclear Physics and Astrophysics, and Department of Physics, University of Washington, Seattle, WA 98195 USA; 51 University of Chinese Academy of Sciences, Beijing, 100049 China; 52 Physics Department, Stanford University, Stanford, CA 94305 USA; 53 Physics Department, Williams College, Williamstown, MA 01267 USA; 54 Physics Department, Princeton University, Princeton, NJ 08544 USA

## Abstract

Spurious-electron signals in dual-phase noble-liquid time projection chambers have been observed in both xenon and argon Time Projection Chambers (TPCs). This paper presents the first comprehensive study of spurious electrons in argon, using data collected by the DarkSide-50 experiment at the INFN Laboratori Nazionali del Gran Sasso (LNGS). Understanding these events is a key factor in improving the sensitivity of low-mass dark matter searches exploiting ionization signals in dual-phase noble liquid TPCs. We find that a significant fraction of spurious-electron events, ranging from 30 to 70% across the experiment’s lifetime, are caused by electrons captured from impurities and later released with delay time constants on the orders of 5 ms and 50 ms. The rate of spurious-electron events is found to correlate with the operational condition of the purification system and the total event rate in the detector. Finally, we present evidence that multi-electron spurious electron events may originate from photo-ionization of the steel grid used to define the electric fields. These observations indicate the possibility of reduction of the background in future experiments and hint at possible spurious electron production mechanisms.

## Introduction

The direct search for Weakly Interacting Massive Particle (WIMP) Dark Matter (DM) is one of the most active areas of research in astroparticle physics. Two key parameters that determine low-mass (<10 GeV/c$^{2}$) DM sensitivity with dual-phase noble-liquid time projection chambers (TPCs) are the minimum detectable recoil energy and the background rate.

Heavy WIMP (>10 GeV/c$^{2}$) interactions in a dual-phase TPC are characterised by a prompt scintillation signal (S1), followed by a delayed signal (S2) produced by ionization electrons. These electrons drift upward in the liquid due to a uniform electric field and are extracted into a thin gaseous layer (gas pocket), where they induce electroluminescence. As discussed in Refs. [1, 2], S1 and S2 have different pulse shapes. In liquid argon (LAr), S1 rises in a few nanoseconds and falls as a double exponential, with mean lifetimes $\tau _1\sim {6}\,\text {ns}$ and $\tau _2=({1.4\pm 0.1}) \,\upmu \text {s}$ [3], and a prompt fraction (light collected in the first 90 ns over total) of approximately 30% (70%) for electronic (nuclear) recoils. This difference in S1 pulse shape enables efficient pulse shape discrimination (PSD) between electronic and nuclear recoils [4]. In the DarkSide-50 configuration (described in Sect. 2), S2 has a characteristic rise-time of $({1.2\pm 0.5})\,{\upmu \text {s}}$ and falls exponentially with a $({3.43\pm 0.06})\,{\upmu \text {s}}$ time constant [2], and lasts for a few tens of  μs. Detecting both S1 and S2 allows for the 3D position reconstruction of interaction vertices, enabling background rejection through fiducialization and multiple-scatter rejection.

Low-mass WIMP searches, focused on <10 GeV/$c^{2}$ masses, have been performed using primarily the ionization signal to detect interesting events (S2-only analysis). While the S1 photon detection efficiency is limited by the detector’s light collection efficiency, extracted electrons generate an S2 signal that can be detected with high efficiency due to the secondary emission of photons in the gas pocket, caused by the acceleration of electrons in the high field region (electroluminescence). In DarkSide-50 this amplification was $(23 \pm 1)\text {PE (photoelectrons)}/\text {e}^{-}$ [5]. As a result, lower energies can be accessed in the S2-only channel than when both S1 and S2 are required. The S2-only approach has been used in several light DM searches [5–11], and it is proposed for future searches for light DM [12, 13] and Coherent Elastic Neutrino-Nucleus Scattering (CEνNS) bursts from supernova neutrinos [14]. S2-only analyses have also been used to search for CEνNS from reactor neutrinos [15] and solar neutrinos [16].

All S2-only analyses, with both argon [5, 6, 17–19] and xenon [7–9, 20–27], have seen excess rates of signals corresponding to a few extracted electrons, herein referred to as Spurious Electrons (SEs), inconsistent with radioactive background models (illustrated and discussed below, in Fig. 17). Their origin is not completely understood, and their presence limits the sensitivity of these experiments. For instance, for the DarkSide-50 S2-only light DM searches [17–19], only events at energies where the SE background was found to be negligible were retained, thus restricting sensitivity to WIMP masses above 1.2 GeV/$\text {c}^{2}$. Understanding the nature of these events is therefore the key to improving the sensitivity of low-mass WIMP searches.

This paper presents the first systematic study of SE events in LAr, using data collected by the DarkSide-50 detector located at LNGS. The findings from this study are compared to similar findings with xenon detectors.

In Sect. 2, we describe the DarkSide-50 detector. Section 3 describes the data selection and classification of event types, including SE selection cuts and event rate trends. These trends are correlated with measures of the detector purity in Sect. 4. Correlations of SEs with other events and with detector parameters are explored in Sect. 5. The electron multiplicity in the SE events is studied in Sect. 6. Finally, Sect. 7 places results in a broader context and discusses possible mechanisms of SEs production/emission, followed by a summary in Sect. 8.

## The DarkSide-50 detector

DarkSide-50 LArTPC has a cylindrical target defined by a 348.8 mm diameter and a 348.8 mm height (at LAr temperature) and consisting of (46.4 ± 0.7)kg of underground argon (UAr) with an ^39^Ar concentration at least 1400-times lower than in atmospheric argon [28, 29]. S1 and S2 photons are detected with two arrays of 19 3” diameter Hamamatsu R11065 photomultiplier tubes (PMTs), which view the UAr from above and below through fused-silica windows. The windows are coated on both faces with 15 nm-thick transparent and conductive indium tin oxide (ITO). These coatings form the TPC’s grounded anode (top) and negative high-voltage cathode (bottom), while their outer faces are held at the mean PMT photocathode potential.

The cylindrical wall is a 2.54 cm-thick polytetrafluoroethylene (PTFE) reflector fabricated with a modified annealing cycle to increase its reflectivity. The TPC’s inner surfaces are coated with tetraphenyl butadiene (TPB), a wavelength shifter that absorbs LAr’s 128 nm scintillation photons and re-emits visible photons (peaked at 420 nm), detected by the PMTs with 35% peak quantum efficiency. Ionization electrons drift towards the LAr surface at (0.93 ± 0.01) mm/μs due to a 200V/cm drift field, with a maximum drift time of 376 μs. An etched 50 mm-thick stainless steel grid positioned 4.7mm (at LAr temperature) below the liquid surface creates a 2.8–3.7 kV/cm extraction field in the liquid above the grid extracting electrons into the gaseous argon (GAr) with >99.9 % efficiency [30] and a 4.2–5.6 kV/cm electroluminescence field in the gas pocket producing S2 via electroluminescence. These variations of the field strengths are caused by local variations in the gas pocket thickness (e.g. due to sagging of anode window) or shape deformation of the grid [31].

To maintain and improve purity, argon is extracted from the cryostat in the gas phase by a circulation pump at a nominal rate of ∼28 SLPM (2.69 kg/h). The GAr passes through a hot getter (SAES Monotorr PS4-MT50-R-2 [32]), before passing through a cold charcoal radon trap, cooled by the GAr. Immediately before entering the cold trap, the inlet GAr is cooled by outlet nitrogen gas from a LAr condenser through a heat-exchanger, decreasing the required cooling power. The temperature of the cold trap is correlated with the GAr flow rate. Condensed LAr is directly injected into the TPC by gravity. See Ref. [33] for the schematic of the DarkSide-50 cryogenic system.

A hardware event trigger occurs when signals in 2 or more PMT channels exceed ∼0.6 photoelectrons (PE) within a 100 ns window. Subsequent triggers are inhibited for 810 μs, and waveform data are recorded from all 38 PMTs for 440 μs starting 10 μs before the trigger. The data reported here were acquired between April 2015 and February 2018.

## Data selection and classification

All triggered events are processed with the same low-level reconstruction software used in Ref. [34]. A pulse-finding algorithm is applied to the digitized data, including the pre-trigger window. Each identified pulse is characterized as S1 or S2 based on a calculated $f_{90}$ parameter, defined as the fraction of light detected in the pulse’s first 90 ns: S1 pulses have $f_{90}$>0.15, whereas slower-rising S2 pulses tend to have lower $f_{90}$ values. The efficiency of the pulse finding algorithm is essentially 100 % for S2 signals larger than 30 PE [34]. No corrections accounting for the trigger and pulse-finding inefficiency are applied.

**Fig. 1:**
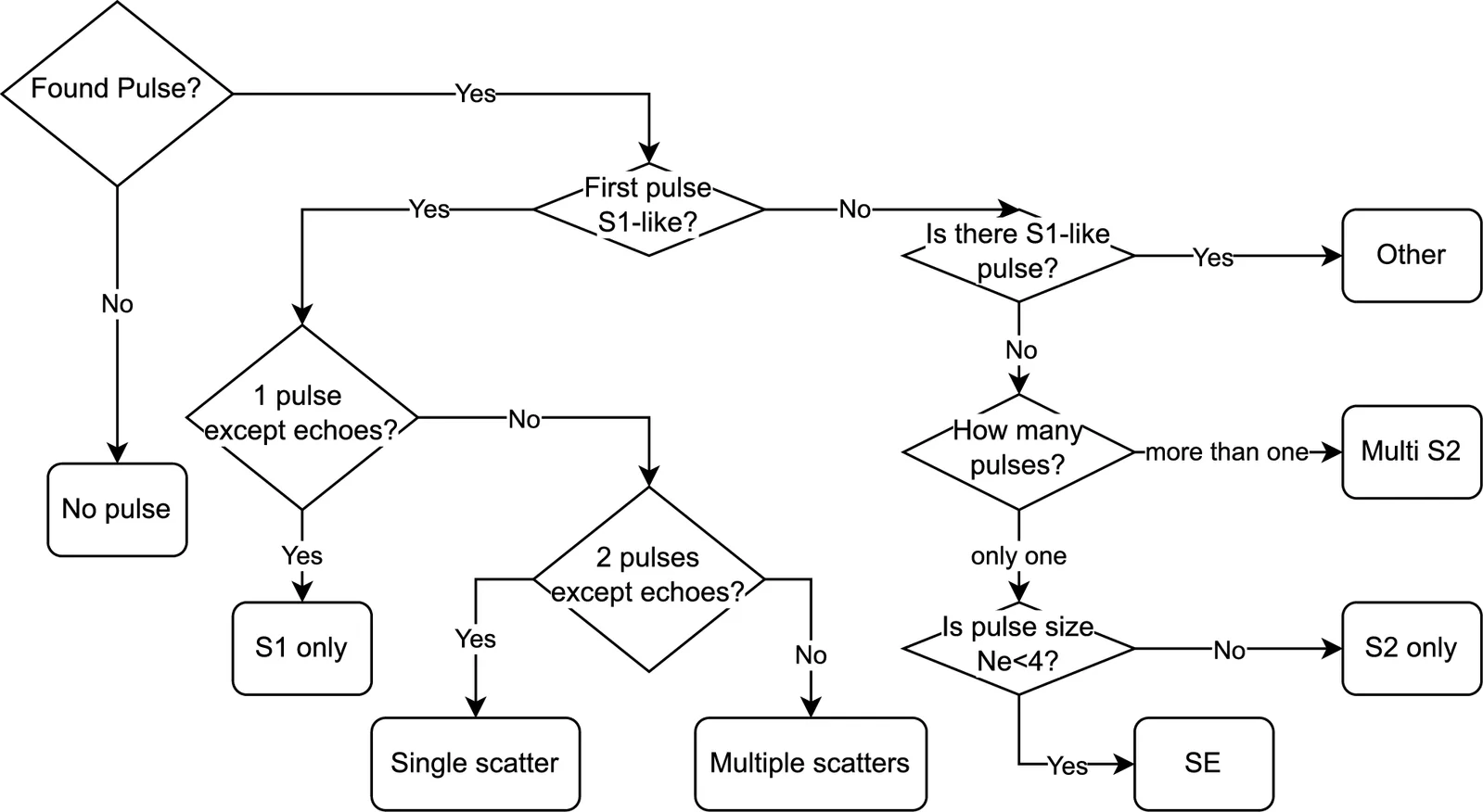
Event categorization scheme. After the basic requirements, all events are categorized in one of those categories

Due to the observed radial variation in electroluminescence yield [5], the trigger and pulse-finding efficiencies for single electron events depend on the events’ radial positions. S2-like pulses are corrected for this variation using the radial position of the PMT channel recording the most light, as in Ref. [6]. After normalizing for PMT gain, this corrected S2 size is referred to as the number of photoelectrons (nPE). For S2-like pulses, nPE is converted to the number of extracted electrons ($N_{e^-}$) by dividing by the single electron light yield under the central PMT: $N_{e^-}=\textsf{nPE}/g_{2}$, where $g_{2}=(23\pm 1)\text {PE}/\text {e}^{-}$.

To characterize SEs, we define an event classification scheme. In the following section, all quoted uncertainties are purely statistical unless otherwise stated.

### Pulse classification

Identified pulses are classified by $f_{90}$ and nPE into two categories: S1-like and S2-like. “Echoes”, S2-like pulses produced by photo-ionization electrons released from the cathode when struck by S1 or S2 photons, are not counted in the total number of pulses. These echoes follow a preceding S1 or S2 pulse by exactly the maximum drift time for electrons in the TPC and are characterized in Ref. [35].

**Table 1:** Summary of event classification categories

Classification	Description	Examples
No pulse	No pulses found	Noise trigger, low $N_{e^-}$ event near walls
S1-only	S1 identified; no S2 found	Cherenkov, surface events
Single-scatter	One S1; one S2 pulse found	Particles scattering once in LAr, S1 pileup
Multi-scatter	One S1; multiple S2 pulses identified	γ-rays scattering multiple times in LAr, random pileup
Multi-S2	No S1; multiple S2 pulses	Multi-scatters with low enough drift time ($t_\textrm{drift}$) or sub-threshold S1
S2 only	No S1; one S2 found with $N_{e^-}\ge 4$	Single-scatter events with S1 below threshold
SE	No S1; one S2 found with $N_{e^-}<4$	Delayed electrons
Other	S2 pulse first, followed by at least one S1 pulse	S2-triggered events with random pile-up

### Event classification

All events are required to satisfy the following three basic requirements as in Ref. [36]: (1) data are present for all TPC channels; (2) baselines for the digitized waveforms are successfully found in all TPC channels; and (3) the event occurs at least 400 μs after the end of the inhibit window of the previous trigger (that is, at least 1.21 ms after the previous trigger). The last requirement removes events whose S1 might have occurred during the inhibit window, and are subsequently triggered by the following S2 pulse.

All events passing these cuts are classified into one of the categories described in Table 1. If the pulse-finder finds no pulses, the event is categorized as “No pulse”, as happens when events are triggered by a small signal that the pulse-finder fails to identify. Events in which at least one pulse is found are separated by whether the first pulse is S1-like or S2-like. Events with an S1-like first pulse are further divided into three categories depending on the number of subsequent S2 pulses: “S1-only” for events with only one pulse, “Single-scatter” for events with two pulses, and “Multiple scatters” for events with more than two pulses. Events with only S2-like pulses are divided into three categories based on the number of pulses: “Multi-S2” for events with more than one pulse, “S2-only” for events with one pulse with $N_{e^-}\ge 4$ (based on the $N_{e^-}$ distribution in Fig. 17), and “SEs” for events with one S2-like pulse with $N_{e^-}< 4$. Events with an S2-like first pulse and at least one S1-like pulse are categorized as “Other”. Figure 1 shows a flow chart for the event classification.

**Fig. 2:**
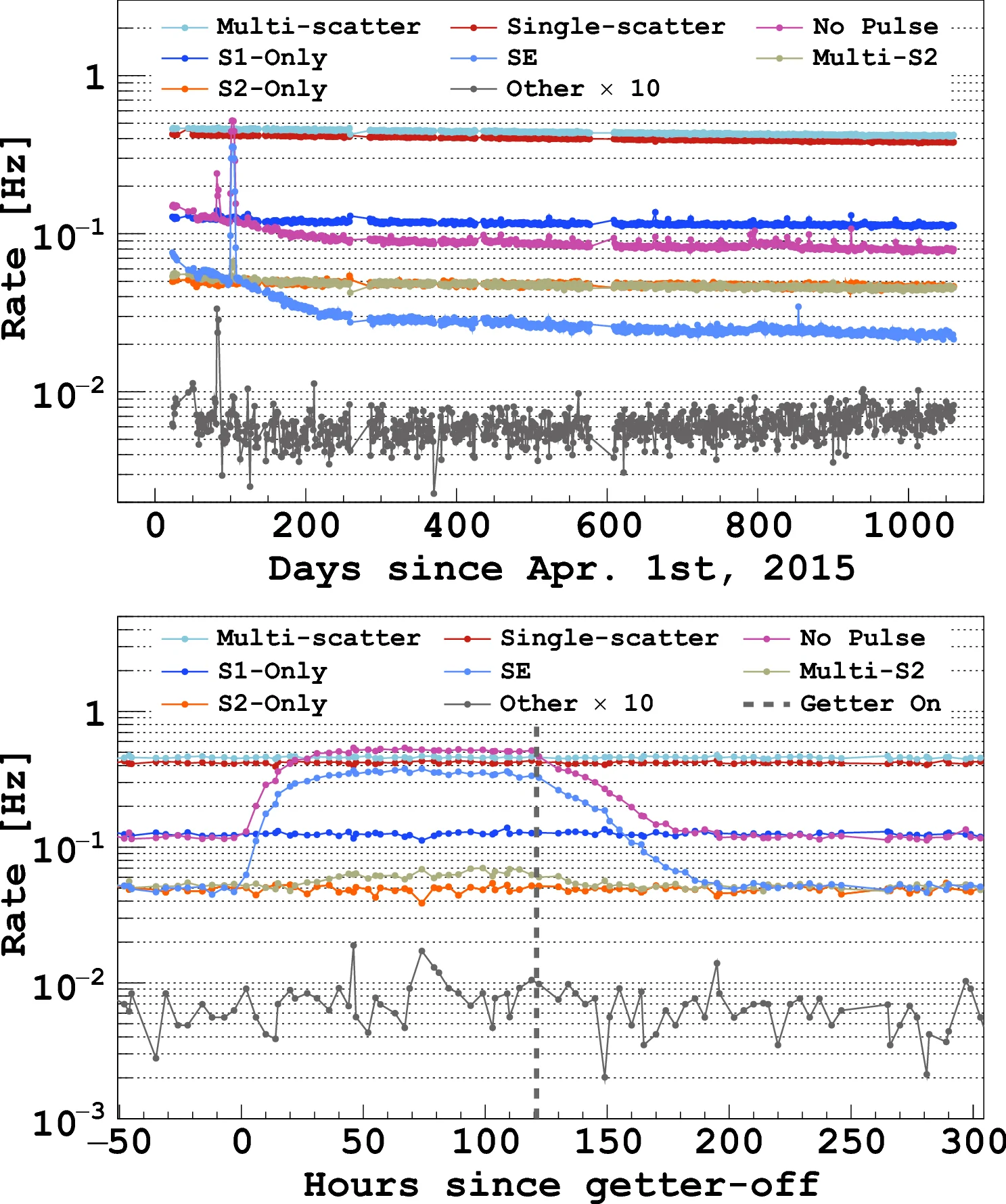
Rate of each event type in data collected with UAr. Top panel shows over the full dataset, and the bottom panel is zoomed in around day 100, showing the time the getter was bypassed (at 0 h) and back on (dashed gray line)

### Time evolution of event rates

The evolution of the event rate of each category, starting from April 1, 2015, the UAr fill date, is presented in the top panel of Fig. 2. Apart from “SE” and “No Pulse” events, each category remains steady over the 1000 days of operation, reflecting the stability of the TPC and cryogenic system. The gaps in time correspond to runs with different configurations, such as those with different field strengths or with calibration sources present. The slight decreasing trends over three years in “Single scatter” and “Multi scatter” events are consistent with the decay of ^60^Co and ^85^Kr. For “SE” and “No Pulse” events, there are two decreasing trends: for the first 200 days, the rate decreases with a ∼65 day decay constant, which then becomes ∼8 years. There are also spikes in “Other” after roughly 80 days, and in the “SE” and “No pulse” categories after roughly 100 days from the UAr fill date. The first was caused by an abnormally high rate in one PMT and is not discussed further in this paper. The latter occurred when the getter was removed from the gas circulation system for maintenance for 5 days starting on day 99.

#### Getter-off period

When the getter was bypassed in the gas circulation system, the TPC event rate increased rapidly. The bottom panel of Fig. 2 shows the rate of each event category as a function of time since the removal of the getter. The rate increase is predominantly due to an increase in the “No Pulse” and “SE” categories, composed of S2-like signals with $$N_{e^-} {<4} $$. There is no corresponding increase in S2-only events with $$N_{e^-} {>4} $$, demonstrating that such events behave differently than other S2-only events. The observed increase in “No Pulse” events is likely a result of misidentified SE events; due to the radial dependence of the S2 yield, an SE-like event near the TPC walls may be under-amplified relative to one near the center of the TPC and fall below the pulse-finder’s threshold. The ∼20% increase in “Multi-S2” event rate (events with no S1-like signals but at least two S2-like signals) is likely due to SEs piling up with each other. The excess event rate increased for 2 days and then stabilized until the getter was re-installed. After reinstallation, the excess event rate decreased with a 36 h time constant, which is in line with the purification rate of ∼20 h to 60 h based on the circulation rate 2.7 kg/h, an active LAr mass of 46 kg in the TPC and a total LAr mass of 155 kg in the cryostat.

## Evolution of purity metrics

The spike in the SE rate when the getter is removed suggests a possible link to impurities in the LAr. Two established measures of the LAr purity are the free electron lifetime $\tau _e$, which measures the fraction of electrons captured by electronegative impurities before reaching the gas pocket, and the lifetime of the long-lived triplet component in S1, $\tau _\ell$, which measures the abundance of impurities that can non-radiatively dissipate energy stored in argon excimers, such as through vibrational degrees of freedom. Although these metrics are not sensitive to all possible impurity types and may have limited sensitivity to dilute impurities, they can still constrain impurities potentially linked to SE production.

### Free electron lifetime

Since electronegative impurities such as ${\textrm{O}_{2}}$ may capture drifting electrons [37, 38], electrons from ionization events closer to the cathode are more likely to be captured before reaching the gas pocket than those produced near the grid. The free-electron lifetime quantifies the average time in which electrons drift before being captured. This is a function of the electronegative impurity concentration and electron drift speed. To evaluate $\tau _e$, the dependence of S2 charge on vertical distance between the interaction site and cathode is measured, using both physics data and a mono-energetic ^83 m^Kr calibration source with a 1.83 h half-life injected in the UAr.

#### Measurements with physics data

During physics data-taking, with no calibration sources present, background γ-ray peaks are available for free electron lifetime determination. The value of $\tau _e$ is estimated from the relationship between drift time ($t_\textrm{drift}$) and the ratio S2/S1, after correcting S2 for horizontal position and S1 for vertical position.

**Fig. 3:**
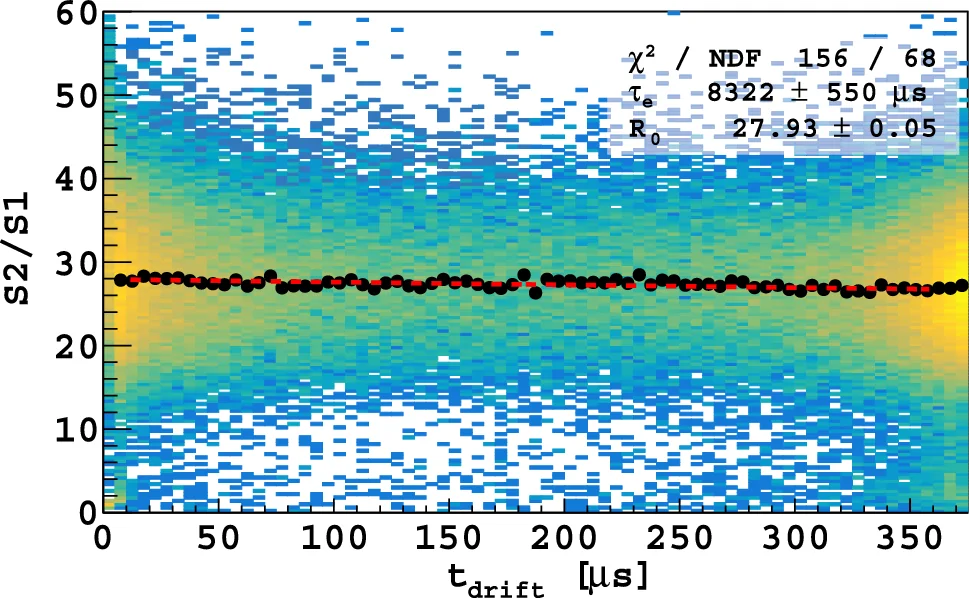
Dependence of S2/S1 on $t_\textrm{drift}$ with the getter off. The black points are mean values in each $t_\textrm{drift}$ bin and the red dashed line is the exponential fit function

After removing the observed energy dependency of S2/S1, the mean value of S2/S1 at a given $t_\textrm{drift}$ is proportional to the fraction of ionization electrons that reach the gas pocket. The observed relationship between S2/S1 and $t_\textrm{drift}$ is shown in Fig. 3 for the runs during the getter off period as an example. A function $f(t_\textrm{drift})=\textrm{R}_0 e^{-t_\textrm{drift}/\tau _e}$ is fit to the mean value of S2/S1, where $\textrm{R}_0$ and $\tau _e$ are fit parameters. The best value of $\tau _e$ obtained in Fig. 3 is 8.3 ms for the getter-off runs.

#### Measurements with ^83 m^Kr calibration data

A ^83 m^Kr calibration source was injected into the UAr approximately annually to study $\tau _e$ and calibrate the S1 light yield. ^83 m^Kr produces mono-energetic signals via sequential decays of 32.1 keV and 9.4 keV, separated with a half-life of 154 ns. To determine the electron lifetime, ^83 m^Kr events are selected around their S1 peak, and mean S2 charge as a function of $t_\textrm{drift}$ is examined, after subtracting the background spectra measured in physics runs as necessary. The mean S2 is then fit as a function of $t_\textrm{drift}$.

**Fig. 4:**
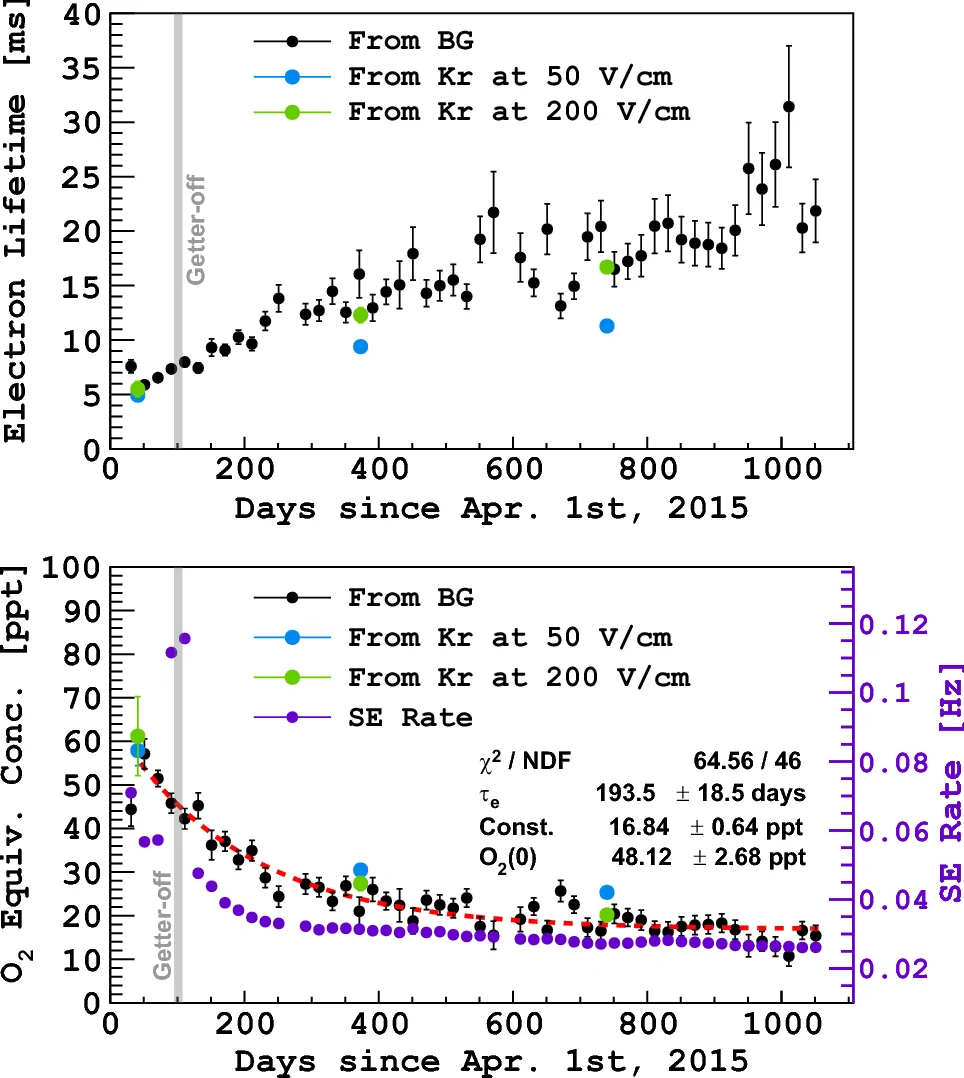
(Top) Electron lifetime vs time (Bottom) ${\textrm{O}_{2}}$ equivalent contamination vs time with time evolution of the total SE rate. The red dashed line shows an exponential function fit to the ${\textrm{O}_{2}}$ equivalent concentration inferred from background (BG) data. The Getter-off days are indicated in gray

The top panel in Fig. 4 shows $\tau _e$ measured in physics and calibration data. For $\tau _e {>10}\,\text {ms}$, the decrease in S2 at large $t_\textrm{drift}$ becomes small, resulting in large statistical uncertainties with a 200 V/cm drift field. To decrease these uncertainties, $\tau _e$ is also measured at a 50 V/cm drift field with the ^83 m^Kr calibration source, where the slower drift speed and higher electron attachment coefficients result in more electron loss [37, 39].

When the getter was removed, $\tau _e$ did not decrease: the measured value of 8.3 ms is consistent before and after this period. The impurities that dominate the electron lifetime therefore cannot alone explain the rapid increase in the SE rate when the getter was turned off.

Taking ${\textrm{O}_{2}}$ as a representative electronegative impurity, the ${\textrm{O}_{2}}$ concentration $[{\textrm{O}_{2}}]$ consistent with $\tau _e$ is shown in the bottom panel of Fig. 4, related by [40],

$$\begin{aligned} [{\textrm{O}_{2}}]/[{Ar}] = 1/(35 k\tau _e), \end{aligned}$$

for Ar concentration [Ar] = 35 mol/L and rate constant $k={9 \times 10^{10}}{\mathrm{L/(mol\,s)}}$ at 200 V/cm and ${10^{11}}{\mathrm{L/(mol\,s)}}$ at 50 V/cm [37, 39]. Fitting an exponential distribution to the change in $[{\textrm{O}_{2}}]$ over time gives a decay constant of ∼194 days. There is no clear correlation between the impurities responsible for the finite electron lifetime and the SE event rate, suggesting that the impurities that dominate electron lifetime are not primarily responsible for SEs. Similar results have been found for LXeTPCs, including LUX and XENON1T [41, 42].

### Pulse shape

S1 pulse shape fits in LAr were performed by WArP [43], DEAP-3600 [44], DUNE [45], and ARIS [46]. WArP studied the pulse-shape dependence on ${\textrm{N}_{2}}$ concentration $[{\textrm{N}_{2}}]$, showing changes in the triplet component’s amplitude $p_\ell$ and lifetime $\tau _\ell$ for $[{\textrm{N}_{2}}]{>1\,\textrm{ppm}}$. This effect is understood to be due to quenching from non-radiative reactions between ${\textrm{Ar}_{2}}$ dimers and ${\textrm{N}_{2}}$ molecules. Since triplet dimers live longer than singlets, they are more heavily suppressed. For $[{\textrm{N}_{2}}]\gtrsim {1\,\textrm{ppm}}$, it can be estimated using $\tau _\ell$ [43]. The impact of ${\textrm{N}_{2}}$ in DarkSide-50 is measured by searching for correlations between $\tau _\ell$ and the SE rate.

To estimate $\tau _\ell$, 50000 waveforms are averaged in 2 day intervals, requiring: (1) $\text {S1}\in [{100},{20000}]\,{\textrm{PE}}$; (2) exactly 2 pulses were identified; (3) $t_\textrm{drift}$>20 μs; (4) ≥20 ms have passed since the previous event; (5) ${0.15} <f_{90}< {0.5}$; (6) and top-bottom asymmetry $A_\text {t-b}\in [{-0.9},{0.9}]$, where $A_\text {t-b}=(\text {PE}_\text {t}-\text {PE}_\text {b})/(\text {PE}_\text {t}+\text {PE}_\text {b})$ and $\text {PE}_\text {t(b)}$ is the number of PE in the top (bottom) PMT array. These criteria select isolated, single-scatter electronic recoils from the bulk LAr, at least 2 cm below the extraction grid.

The average waveform is fit using the function *F* described in Sec. 3.1 of Ref. [46];

1 $$\begin{aligned}&F(t|p_\text {TPB},p_s,p_\ell ,\tau _s,\tau _\ell ,\sigma ,t_0,A,C) = C + A\nonumber \\&\quad \times \sum _{i=\{s,\ell \}}p_i\left[ (1-p_{_\text {TPB}}) P_0(t^\prime | \tau _i,\sigma )+p_{_\text {TPB}} P_1(t^\prime |\tau _i,\tau _{_\text {TPB}},\sigma )\right] ,\nonumber \\&\text {where }t^\prime =t-t_0,\nonumber \\&P_0(t^\prime |\tau _i,\sigma ) = \frac{1}{2\tau _i}\left( 1+\text {erf}\left( \frac{t^\prime -\sigma ^2/\tau _i}{\sqrt{2}\sigma }\right) \right) e^{\frac{-t^\prime }{\tau _i}+\frac{\sigma ^2}{2\tau _i^2}},\nonumber \\&P_1(t^\prime |\tau _i,\tau _\text {TPB},\sigma )=\frac{\tau _iP_0(t^\prime |\tau _i,\sigma )-\tau _\text {TPB}P_0(t^\prime |\tau _\text {TPB},\sigma )}{\tau _i-\tau _\text {TPB}}. \end{aligned}$$

**Fig. 5:**
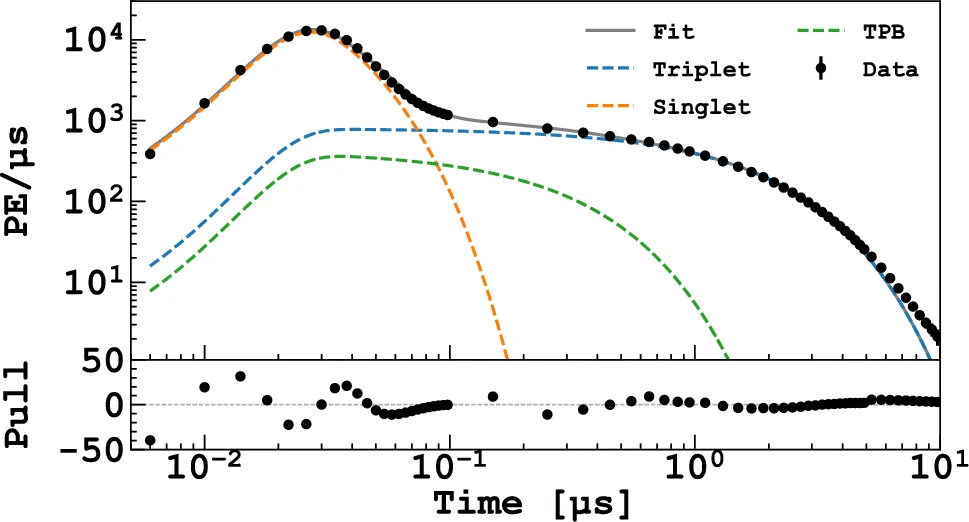
Example average waveform. (Top) Fit with *F*(*t*) from Eq. 1 (gray), with contributions from the singlet (orange), triplet (blue), and TPB (green) components shown. (Bottom) Pulls from the fit, (Data-Func.)/Error of Data

This model describes two exponential decays with probabilities $p_s$ and $p_\ell$ and lifetimes $\tau _s$ and $\tau _\ell$ corresponding to the short-lived (singlet) and long-lived (triplet) components. This distribution is convolved with the TPB’s re-emission modeled with a prompt and a delayed component, and a Gaussian distribution with timing offset $t_0$ and resolution σ. The present analysis differs from Ref. [46] in two ways: (1) only one TPB component is considered, and (2) a constant term *C* is fixed to 0. Both differences have negligible effects on the present study. Figure 5 shows one such fit, performed from the start of the waveform to 10 μs.

Based on the fit results, no correlation is observed between $p_\ell$ or $\tau _\ell$ with the SE rate, including the getter-off period. At the end of the data set, where the electron lifetime is longest, the triplet lifetime $\tau _\ell$ is $({1.377\pm 0.008})$ μs, which is consistent with Refs. [44, 45]. Throughout the data collection period, $\tau _\ell$ decreases by $<{0.050}$ μs (90% CL). Allowing *C* to be nonzero in these fits increases $\tau _\ell$ by 4%; also accounting for the second TPB-re-emission component increases $\tau _\ell$ by 14%. Varying the fit range from 5 μs to 15 μs increases $\tau _\ell$ by up to 3%. No energy-dependence of $\tau _\ell$ is observed within the considered PE range.

Based on these results and those in Ref. [43], the increase of SE rate during the getter-off period is not linked to increased ${\textrm{N}_{2}}$ contamination above the 1 ppm level.

## Observed spurious electron correlations

Several mechanisms have previously been proposed to explain the SEs observed in liquid xenon TPCs, including chemical impurity interactions, delayed electron extraction into the gas phase [22], spontaneous field emission of the grid, and de-excitation of metastable target excimers [7, 22, 24, 41]. Many of these mechanisms may also be applicable to LAr TPCs and can be studied by examining the correlations between SEs and preceding events.

A subset of “Single-scatter” and “Multi-scatter” events that may be followed by SEs, called “parent events” (or simply “parents” in the following), are selected for the study of SE event correlations. Unless otherwise specified, they are required to have $\text {S1}{>1000\,\mathrm{\textrm{PE}}}$ after correcting for the vertical position, one S1, at least one S2, and a successfully-reconstructed horizontal position. This S1 threshold is chosen to increase the probability of the parent being followed by SEs, as will be discussed later.

### Time correlation with previous events

To study coincidences between SEs and parents, the time difference $T_{diff}$ separating each SE from all preceding parent candidates in a 10 s window (time-ordered pairs) is calculated. The contribution of random coincidences is estimated using $T_{diff}$ for parents that follow SEs in the same time span (random pairs). Values of $T_{diff}$ are binned in a histogram with logarithmic bin widths, reflecting the correlation’s exponential nature. Due to the inhibit time and veto windows used for this analysis, these fits are not sensitive to correlations on <1 ms timescales. Much shorter correlations, with <370 μ s delays, are discussed in Ref. [35], which explores effects such as photoelectric emissions.

**Fig. 6:**
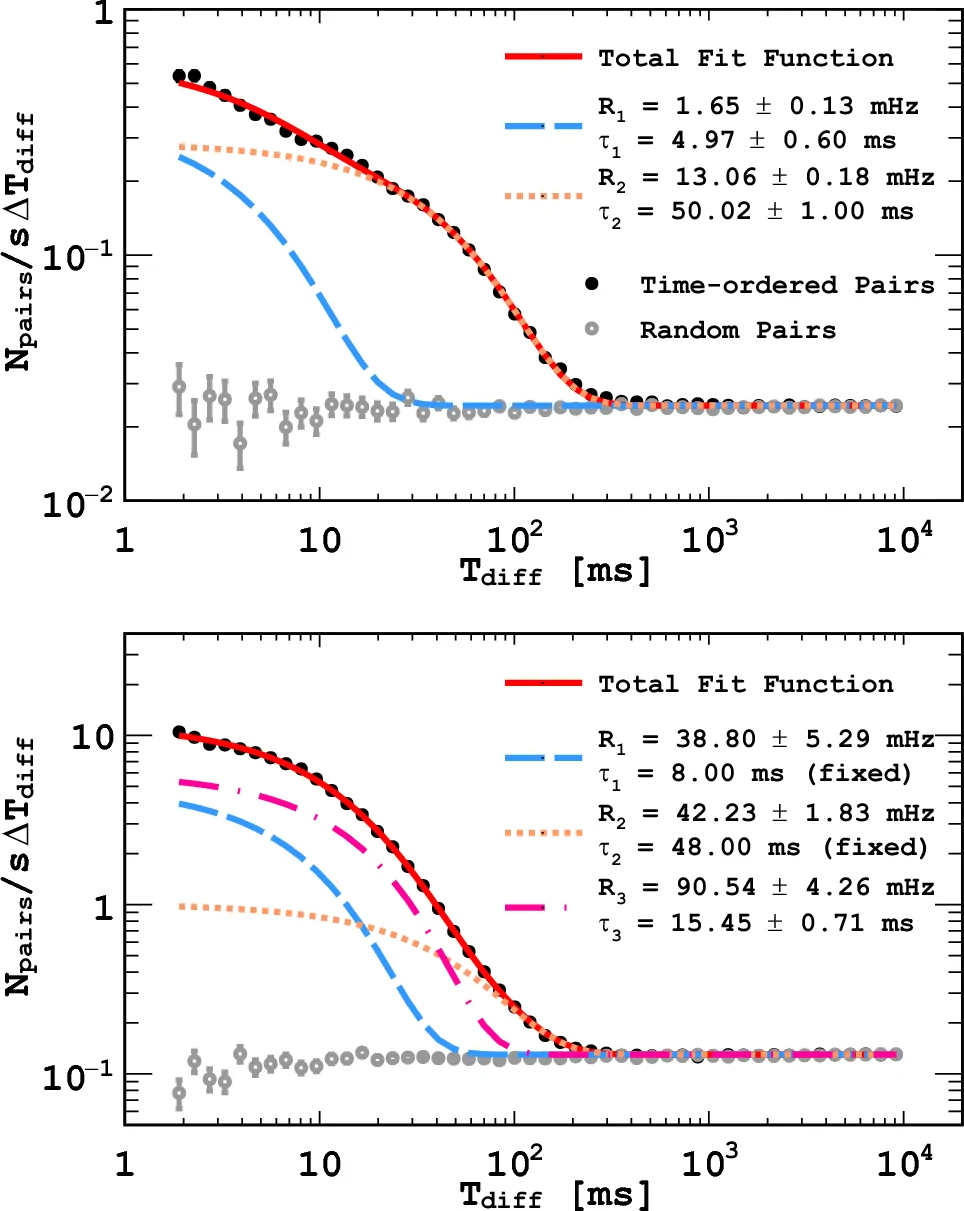
The time difference $T_{diff}$ separating SEs and all preceding parent events in a 10 s window, normalized to lifetime and bin width. Closed black circles show SEs following parents (time-ordered pairs), while open gray circles show random pairs, which have a flat distribution as expected. The red solid line shows the total fit function, with two exponential components and a constant. (Top) Shows physics data from runs 319–353 days after the UAr fill, fitted with two exponential components (dashed blue and dotted orange lines) and a constant. (Bottom) Shows data with the getter off, fitted with two exponential component with fixed τs from fit just before the getter off period, an additional free exponential component (dash-dotted pink line), and a constant. $\Delta T_{diff}$ is the bin width of x-axis

**Fig. 7:**
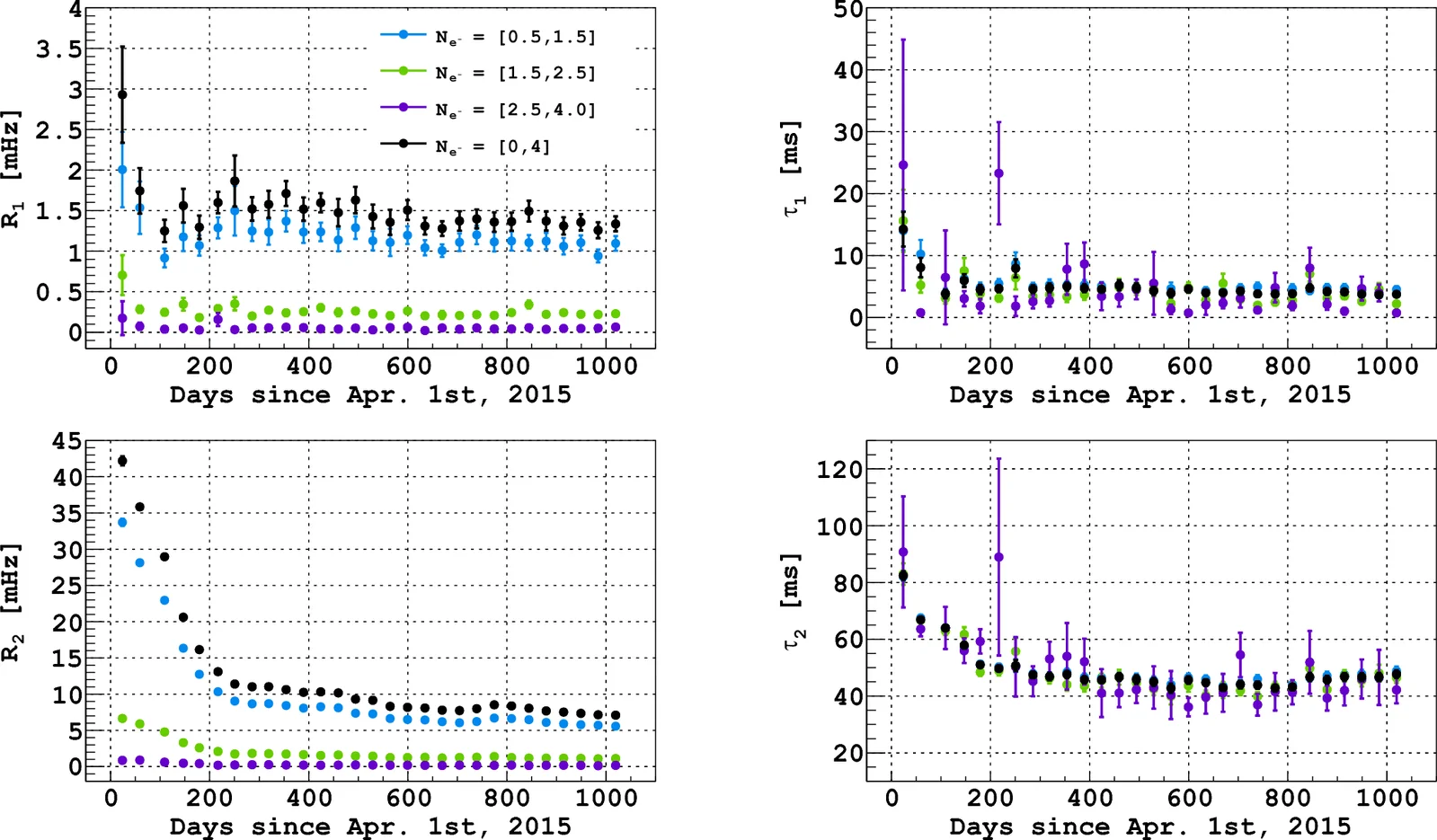
$T_{diff}$ fit parameters for various $N_{e^-}$ ranges, excluding runs for which the getter was turned off

Runs were grouped in 35 day intervals, excluding runs with the getter off. The average rate of SE-parent pairs in each $T_{diff}$ bin is calculated by dividing the number of pairs by the livetime of the 35 day interval. The resulting $T_{diff}$ distribution, after normalizing by the width of each $T_{diff}$ bin ($\Delta T_{diff}$), is well-described by the sum of two exponential functions:

2 $$\begin{aligned} f(T_{diff}) = \frac{R_1}{\tau _1}e^{-T_{diff}/\tau _1}+\frac{R_2}{\tau _2}e^{-T_{diff}/\tau _2} + C, \end{aligned}$$

where $R_1$ and $R_2$ are the rates of two components with decay constants $\tau _1$ and $\tau _2$, with $\tau _1 < \tau _2$, and *C* accounts for random coincidences. For data with the getter off, an additional component with an intermediate decay constant is needed to describe the data.

The top panel of Fig. 6 shows the $T_{diff}$ distribution observed over a 35 day period starting 319 days after the UAr fill. Equation 2 is fit to this distribution, showing strong agreement between data and this decay model. With the getter off, a component with a 15 ms decay constant appears, as shown in the bottom panel of Fig. 6, with $\tau _1$ and $\tau _2$ fixed with their values before the getter-off period. Reduced $\chi ^2$ values for these fits are between 0.9 to 1.7.

Figure 7 shows the results of the fits to $T_{diff}$ distributions, performed for 35-day ranges during the ∼1000-day data collection period. During each interval, events were analyzed separately according to their $N_{e^-}$ values. These fits show that $R_1$ and $\tau _1$ are stable near 1.5 mHz and 5 ms, respectively. However, $R_2$ and $\tau _2$ decrease significantly during the first 200 days and continuously decrease afterwards at a slower rate: $\tau _2$ decreases from 80 ms to 40 ms, and $R_2$ decreases from 43 mHz to 10 mHz.

Temporal variations in $\tau _1$ and $\tau _2$ are independent of $N_{e^-}$, potentially indicating that SEs with different $N_{e^-}$ share a common origin.

**Fig. 8:**
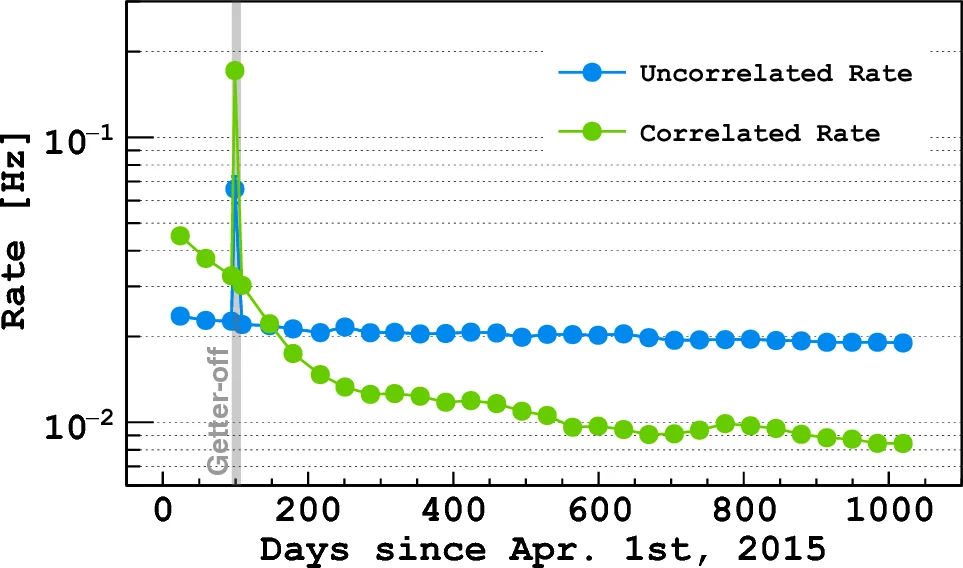
Rate of correlated SE events (green), found by summing the $R_1$ and $R_2$ fit parameters. Rate of remaining uncorrelated events is plotted in blue. Days with the Getter-off are indicated in gray

The sum $R_1+R_2$ gives the total rate of SEs with an identified temporally-correlated parent. Note that this temporally correlated rate may be slightly underestimated due to missing parents that fall outside the parent selections or within the inhibit-time window. Figure 8 compares the correlated SE rate to the uncorrelated SE rate, defined as the remainder of the total SE rate from Fig. 2 after subtracting $R_1+R_2$, consistent with SEs with no identified parent. While the correlated SE rate decreases significantly in the first 200 days and then more slowly, the uncorrelated rate remains approximately constant while the getter was in use. When the getter was bypassed, the rate of correlated SEs increased 6-fold, while the uncorrelated SE rate tripled. At the start of data collection, the correlated component accounts for approximately 70% of all SEs, decreasing to 30% after 200 days.

Given the low probability of seeing SEs from low-energy parents (as will be discussed in Sect. 5.3), changing the parent threshold from $\text {S1}>{1000\,\mathrm{\textrm{PE}}}$ does not significantly change the fraction of correlated events. Specifically, lowering the parents-selection threshold to $\text {S1}>{50\,\mathrm{\textrm{PE}}}$ increases the correlated component fraction by only 2%.

**Fig. 9:**
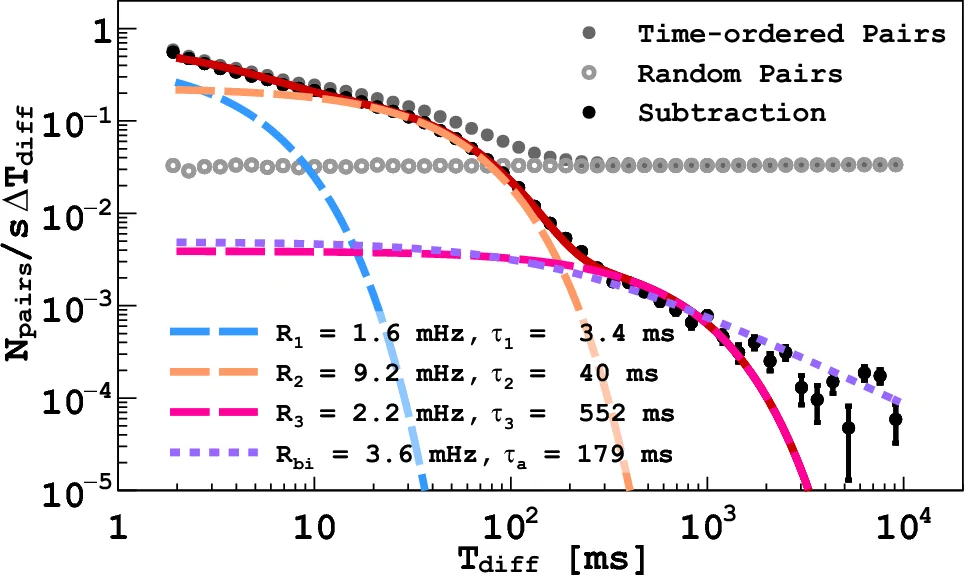
The time difference $T_{diff}$ observed over 500 days. The black solid points are the difference of time-ordered pairs from random pairs with a three-exponential function fit in a red solid line. The blue, orange, and pink dashed lines show the three exponential components, respectively. The purple dotted line shows the biexcitonic ionization function. $\Delta T_{diff}$ is the bin width of x-axis

Figure 9 shows the same analysis with runs grouped into longer time intervals, to increase statistics at larger values of $T_{diff}$. The observed $T_{diff}$ distribution is selected from a late 500 day-period where $\tau _{1,2}$ and $R_{1,2}$ are stable. The data suggest the existence of a subdominant long-lived component not described by the two-exponential model. A fit with the sum of three exponential functions (a simple extension of Eq. 2) returns a decay constant around $({552\pm 33})$ ms for the third exponential function, constituting ∼7% of all SEs. Figure 9 suggests that a longer-lived component $(T_{diff} > 1 s)$ may behave like a power law at late times but is subdominant at earlier times. This behavior may also be a result of exponential contributions that vary over time, obfuscated by low statistics. However, as shown in Fig. 9, the $T_{diff}$ distribution is also well-described by a third component that evolves as $dR_{bi}/dt = N_{bi}/(1+t/t_a)$, where $N_{bi}$ and $t_a$ are constants. This function can describe the decay rate of a long-lived state via biexcitonic ionization processes, which decrease with a time scale $t_a$ due to diffusive processes, and is consistent with the functional form derived in Refs. [47, 48] and explored in Ref. [49] in the limit where the diffusing species are long-lived and sparse. This function was fit to the data along with two exponential functions by fixing their parameters to the result of the three-exponential function fit. The best-fit parameters for $dR_{bi}/dt$ allow this component to describe about 12% of all SEs.

S2-only events with $N_{e^-}>4$ do not show temporal correlations with preceding events in a 1 s window, indicating that the observed correlation is particular to SEs.

### Correlation with total ionization activity

As shown in Fig. 8, about half of SEs are described by correlated components $R_1$ and $R_2$, which vary over time. This method of identifying correlations loses sensitivity for correlation timescales that are long compared to the total 1.5 Hz event rate. SE production mechanisms related to parents may therefore not be counted as “temporally correlated” if their delay is too long.

**Fig. 10:**
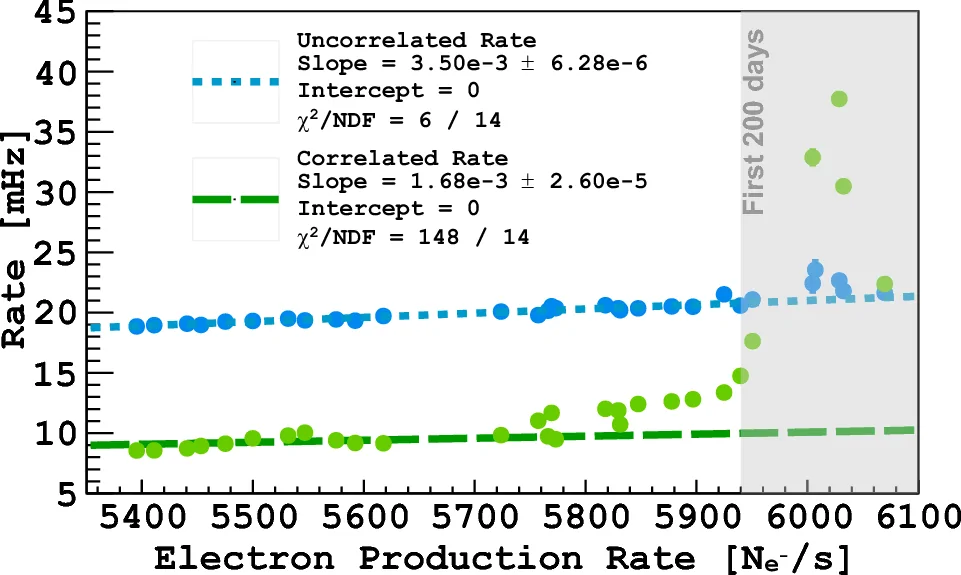
The rate of correlated (green) and uncorrelated SEs (blue) as a function of total electron production rate in 35 day intervals excluding getter-off runs. Linear fits are performed on runs collected after the first 200 days. The shaded area indicates data from the first 200 days

Figure 10 shows the SE rate averaged over 35 day intervals as a function of the average ionization rate, measured as the total $N_{e^-}$ in all non-SE S2 pulses. The correlated SE rate from a single impurity is proportional to the ionization rate; however, since SEs have multiple sources that vary in intensity (e.g., impurities that are removed over time), the final relationship is not strictly linear. Since uncorrelated SEs may not be related to energy depositions in the LAr, such proportionality is not guaranteed – a constant SE production rate may be present in the limit where no ionization events occur in the LAr. This relationship is explored in Fig. 10 by a linear fit with some *y*-intercept corresponding to the SE rate expected with no ionization within the TPC, and a slope describing SEs related to ionization events.

After the first 200 days, this trend is well-described by a linear fit with the intercept fixed to 0, implying that the data are consistent with all apparently-uncorrelated SEs actually having an undetected parent. Varying the intercept value and repeating the fit show that uncorrelated SEs have a rate <7.5 mHz (90% CL). The uncorrelated rate in Fig. 8 is approximately 20 mHz, meaning that less than a third of this component can be independent of total TPC ionization activity.

### Energy correlation with previous events

To study the energy dependence of SEs, they are paired with the most recent single-scatter parent candidate, relaxing the parent identification threshold to $\text {S1}{>50\,\mathrm{\textrm{PE}}}$. Given that the rate of parent events is ∼1.5 Hz and $\tau _2{\sim 50}\,{ms}$, this procedure is robust in identifying the correct SE parent. Based on the fit to the SE timing distribution in Fig. 9, the fraction of mismatched parents is estimated to be at most $\sim 0.5\%$.

**Fig. 11:**
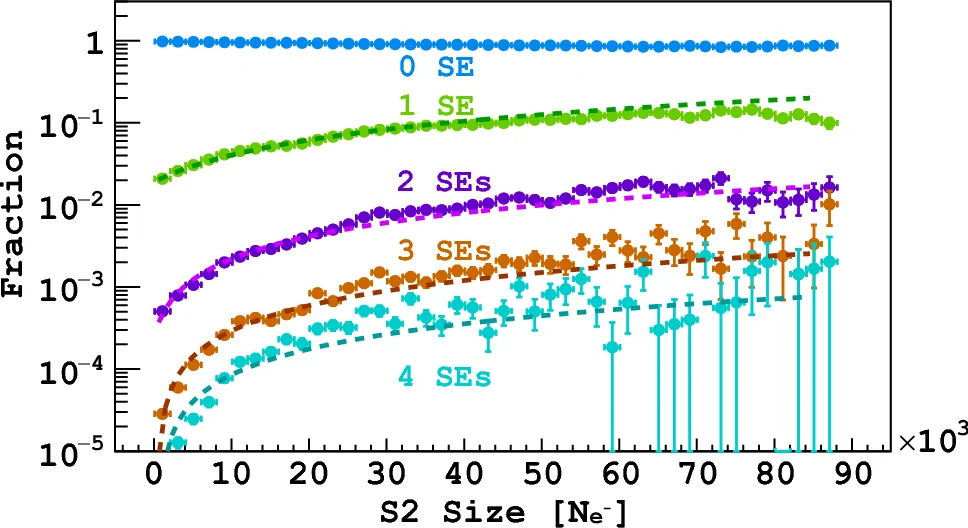
Fractions of parent events followed by different numbers of SE events as a function of the S2 size of the parent. The dashed lines show linear fits

Figure 11 shows the fraction of parents followed by 0–4 SE events before the occurrence of the next parent as a function of its S2 size. The number of SEs following a parent event increases linearly with the parent’s S2, consistent with the hypothesis that SEs are produced by drifting ionization electrons.

**Fig. 12:**
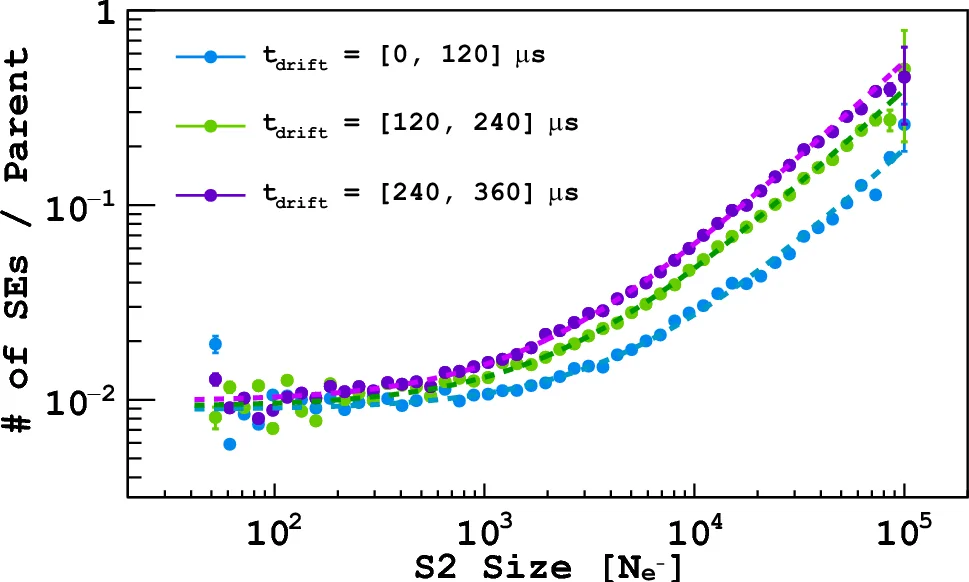
Number of SE events following parent events divided by the number of parent events with a given S2 size. Colors represent cuts in $t_\textrm{drift}$ of parent events. Dashed lines show linear fits

Figure 12 shows the average number of SEs following a parent event, the ratio of the number of SE events to parent events, as a function of parent S2 charge $\text {S2}_\text {parent}$. If SEs are produced with some constant probability per parent electron, this ratio should approach zero for no $\text {S2}_\text {parent}$, and increase linearly with $\text {S2}_\text {parent}$. However, for low-energy parent events, the SE probability approaches a constant value, corresponding to a component of SEs seemingly uncorrelated with parent energy. This component may represent parent-SE pairs where the given SE event originated from some parent other than the one immediately preceding it, caused by a long-lived correlated component (as described in Sect. 5.2). The expectation value of the number of SEs following a parent event $p_{SE}$ can be modeled with

$$\begin{aligned} p_{SE}(\text {S2}_\text {parent}) = c_1 + c_2 \cdot {\text {S2}_\text {parent}}, \end{aligned}$$

where $c_1$ and $c_2 \cdot \text {S2}_\text {parent}$ describe uncorrelated and correlated SEs, respectively. Best-fit values of $c_1$ and $c_2$ are shown in Table 2. At low energies, SEs are dominated by the uncorrelated component, produced at a constant rate. As $\text {S2}_\text {parent}$ increases, correlated SEs become more likely, and $p_{SE}$ scales linearly with $\text {S2}_\text {parent}$.

**Table 2:** Fit parameters to SE event probability as a function of parent S2 size, as plotted in Fig. 12

$t_\text {drift}$ [μs]	$c_1$	$c_2$ [$1/N_{e^-}$]
[0, 120]	$(8.97 \pm 0.13)\times 10^{-3}$	$(1.85 \pm 0.03)\times 10^{-6}$
[120, 240]	$(9.23 \pm 0.19)\times 10^{-3}$	$(3.82 \pm 0.06)\times 10^{-6}$
[240, 360]	$(9.82 \pm 0.14)\times 10^{-3}$	$(5.34 \pm 0.05)\times 10^{-6}$

Figure 12 also shows that the SE event probability depends on $\text {S2}_\text {parent}$ for different ranges of $t_\text {drift}$ of parent events. For low-energy parents, with $\text {S2}\lesssim {10^{3}}e^{-}$, the SE event probability varies little with $t_\textrm{drift}$: $c_1$ increases by 10% as $t_\textrm{drift}$ increases from the lowest to highest interval shown in Table 2. As correlated SEs become more prominent at higher $\text {S2}_\text {parent}$, the SE probability increases with $t_\textrm{drift}$, as $c_2$ increases by a factor of 2.9.

### Drift time correlations with previous events

In order to study spatial correlations between SE and parent events, parent event selections are tightened to ensure that event positions are well-defined by requiring parent events to be single scatter events (one S1 and one S2).

**Fig. 13:**
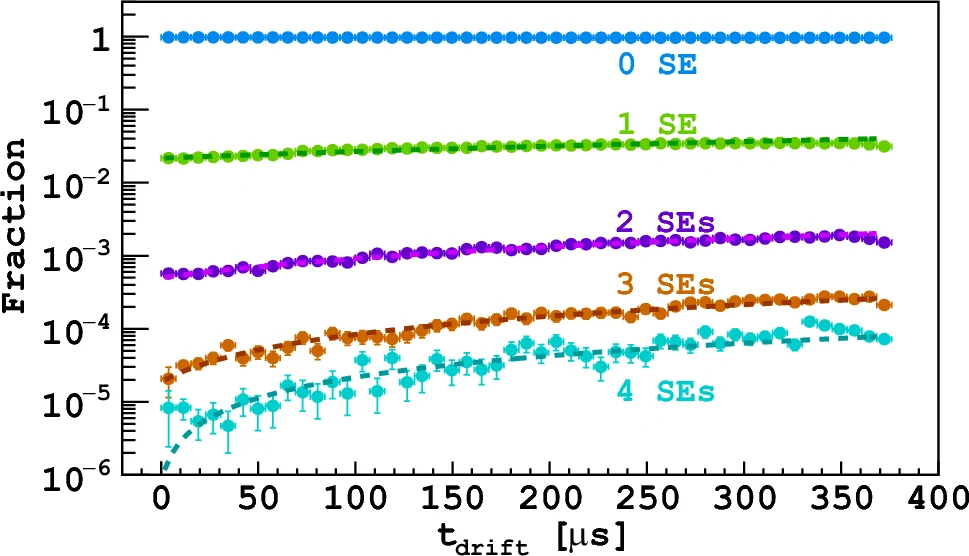
Fractions of parent events followed by different numbers of SE events as a function of parent $t_\textrm{drift}$. The dashed lines show linear fits

To examine the relationship between parent $t_\textrm{drift}$ and SE probability, the fraction of parents followed by an SE is measured as a function of $t_\textrm{drift}$, summing all SEs that follow the parent until the occurrence of the next parent. Figure 13 shows that the expected number of SEs increases with longer parent $t_\textrm{drift}$, as electrons drift greater distances.

**Fig. 14:**
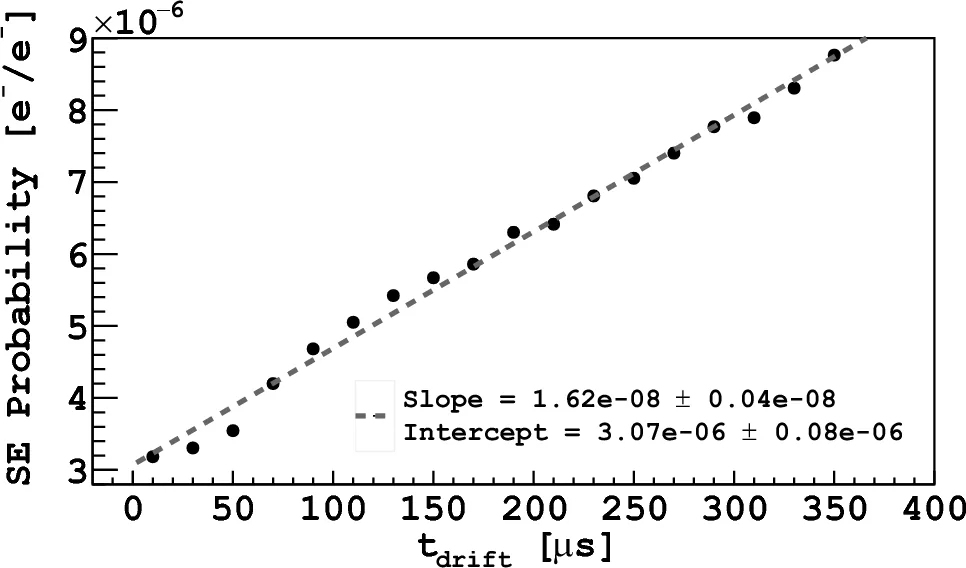
Total charge of all SEs following parent events, normalized by the $\text {S2}_\text {parent}$, as a function of parent $t_\textrm{drift}$

Figure 14 shows the SE charge probability as a function of parent $t_\textrm{drift}$. Normalizing for the parent size, the SE charge probability is estimated as $\sum _i\text {S2}_{\text {SE},i}/\text {S2}_\text {parent}$. Note that it is calculated per unit parent charge rather than per parent event. The relationship between $t_\textrm{drift}$ and SE charge probability is well-described by a line, with slope governed by correlated SEs and *y*-intercept by uncorrelate SEs.

The linear fit gives an SE probability per unit $t_\textrm{drift}$ of $(1.62 \pm 0.04) \times 10^{-8}\,e^{-}/e^{-}/\upmu \text {s}$. With a (0.93±00.1) $\text {mm}/\,\upmu \text {s}$ drift speed, this value corresponds to an SE probability per unit drift length of $(1.74 \pm 0.04)\,\times \,10^{-8}\,e^{-}/e^{-}/\text {mm}$. Notably, the ratio of SE electrons to parent S2 electrons is far smaller than the number of electrons lost by the finite free electron lifetime, discussed in Sect. 4.

### Horizontal position correlation with previous events

To investigate if temporally-correlated SE-parent pairs are spatially correlated, the distance between SEs and parents in the horizontal plane is studied using the same transverse position reconstruction algorithm as in Ref. [36].

**Fig. 15:**
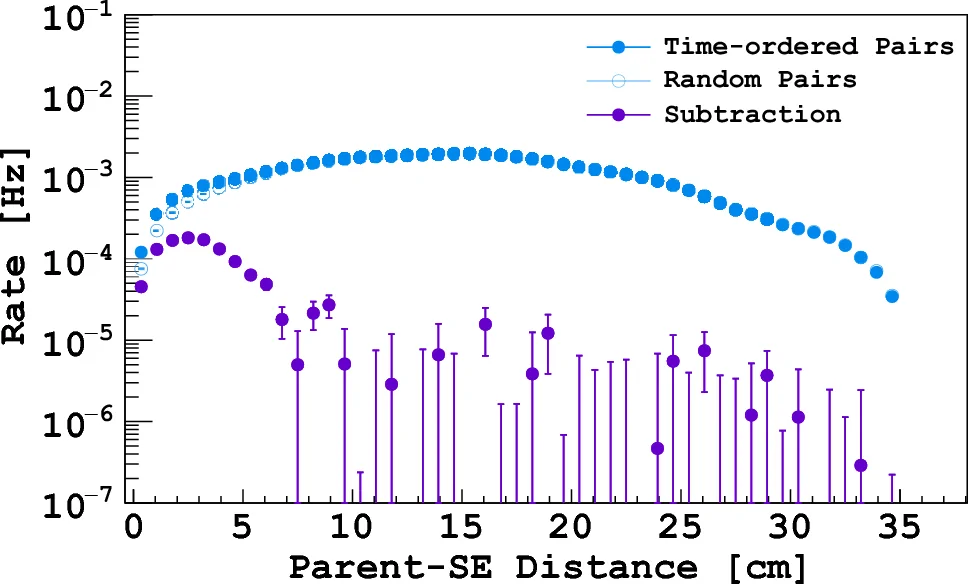
Rate of parent-SE pairs with a given horizontal distance, for (closed circles) correlated and (open circles) random pairs. Purple points result from subtracting random from correlated pairs

Figure 15 shows the reconstructed horizontal distance between all time-ordered SEs-parents pairs within a 10 s window, compared to that for random SE-parent pairs. After subtracting the distribution of the random pairs from the time-ordered pairs, the temporally-correlated pairs predominantly reconstruct within 5 cm of each other. These trends suggest that temporally-correlated pairs are also correlated in their position in the horizontal plane.

### Correlation with monitored hardware parameters

Correlations between the SE rate and monitored detector parameters were studied to investigate the relationship between SEs and the cryogenic and purification systems’ operating conditions.

**Fig. 16:**
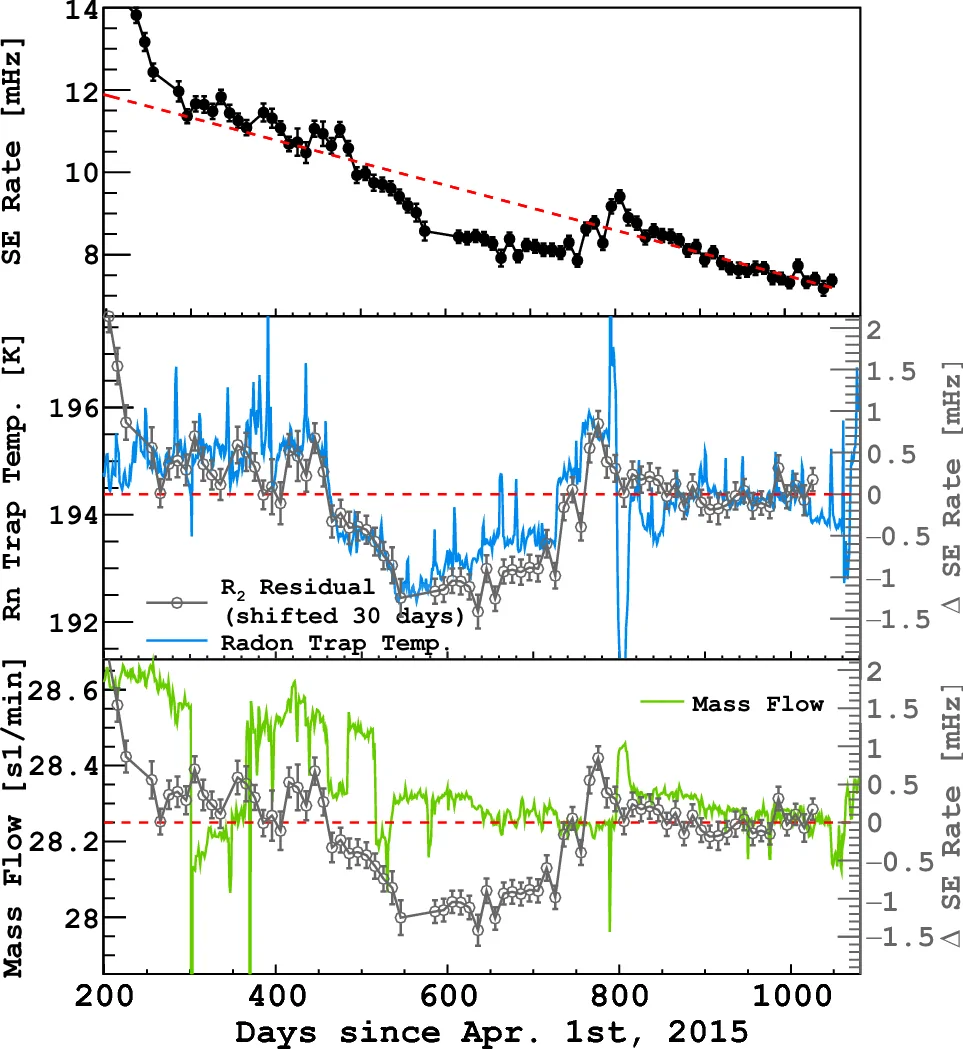
Top: (black) Evolution of the SE rate from the slow component $R_2$, fit with a (red) linear function. Middle: (blue) radon trap temperature compared with (gray) the difference between the SE rate and the linear fit, shifted by 30 days. Bottom: (green) the argon mass flow rate of the circulation, compared with (gray) the same residual rate. Dashed red lines are extrapolated from the linear fits over the stable period between 860–1060 days

Figure 16 shows the evolution of $R_2$ over time, along with deviations from a linear fit compared to the radon trap temperature and the circulation line mass flow, including the hot getter and radon trap. While no correlations were seen for $R_1$, deviations of $R_2$ and the radon trap temperature correlate with each other, offset by approximately 30 days. No clear correlation is seen for the mass flow. Since downward fluctuations in radon trap temperature correlate with lower $R_2$ and, for most molecules, charcoal radon traps capture more molecules at lower temperatures, these observations may indicate that the trap plays a role in the SE event rate. No other parameters showed an obvious correlation with the SE rate change and the mass flow rate through the hot getter and radon trap. The 30 day offset is possibly related to a breakthrough time (the time it takes for a substance to traverse the radon trap) of an impurity that is responsible for the $R_2$ component in the radon trap.

## Electron multiplicity in SE events

Characterizing SEs’ $N_{e^-}$distribution is important for predicting SE backgrounds in future experiments [12, 13].

**Fig. 17:**
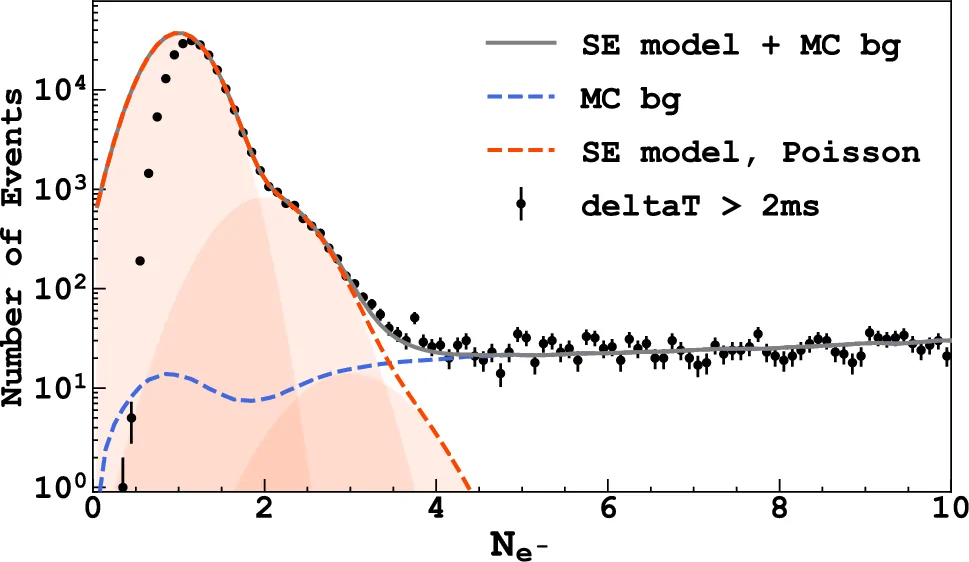
$N_{e^-}$ distribution fit with Monte Carlo simulations of electronic recoil backgrounds (“MC bg”, dashed blue) and SEs (dashed orange), modeled as a Poisson distribution convolved with a Gaussian (pink shaded regions for n=1,2,3)

Figure 17 shows the reconstructed $N_{e^-}$ distribution observed at low energies, compared to Monte Carlo simulations of the radiological background model described in Ref. [5]. Above 4 $\text {e}^{-}$, the distribution is well-described by the electronic recoil background model, while it is dominated by SEs below. The SE distribution is fit by a Poisson distribution describing the amplitude of each *n*-electrons peak, which is modeled as a Gaussian distribution with variance scaling with the number of true extracted electrons *n*:

3 $$\begin{aligned} \text {SE}(N_{e^-})= &   A_\text {SE}\sum _{n=1}^{4} \text {Poisson}(n;\mu _\text {SE})\times \text {Gaus}(N_{e^-};n,\sigma ),\nonumber \\ \sigma= &   \sqrt{n}\sigma _{\text {S2}}, \end{aligned}$$

where σ is the electron-counting resolution, parameterized by $\sqrt{n}$ and the resolution of the 1 $\text {e}^{-}$ yield, $\sigma _{\text {S2}}$. $A_\text {SE}$ is an overall normalization factor. $\mu _\text {SE}$ is the expected number of electrons that reconstruct in the same pulse as an SE, and the characteristic parameter of the Poisson distribution. The model is fit to the data starting at $N_{e^-}$ =1.2, above which the electron tagging efficiency approaches 100%. This fit converges with reduced $\chi ^2= 152/134$, with $\mu _\text {SE}= {0.062\pm 0.001}$ and $\sigma _{\text {S2}}= {0.335\pm 0.001}$. The expected number of electrons in an SE event is given by calculating the expected number of electrons from the Poisson probability without the n=0 case, $\mu _\text {SE}/(1-e^{-\mu _\text {SE}})=1.031$. The same fits are performed for different slices of $T_{diff}$, the time difference between parent and SE events. The fits confirm within statistical uncertainties that $N_{e^-}$distributions are consistent over different $T_{diff}$ bins, including during the getter-off period.

**Fig. 18:**
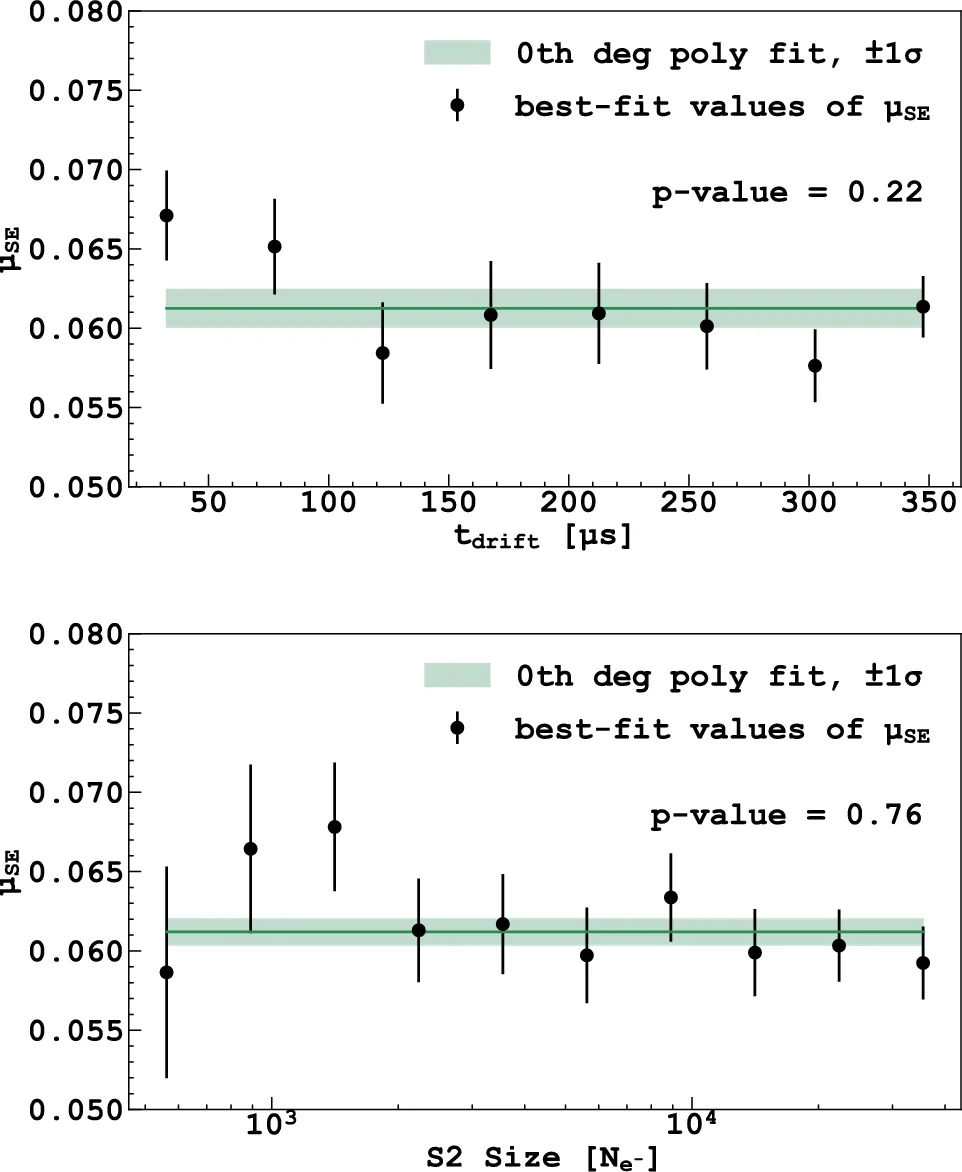
Stability of $\mu _{SE}$. The best values of $\mu _{SE}$ vs. parents’ $t_{drift}$ (top) and vs. parent S2 size (bottom) with constant-value fits and their $\pm 1\sigma$ bands in green

Therefore, $\mu _\text {SE}$ does not depend on the impurity species causing SE events. The variation of $\mu _\text {SE}$ as a function of parent drift time and S2 size was also checked and is shown in Fig. 18. No significant variation in $\mu _\text {SE}$ with respect to parent $t_\textrm{drift}$or S2 size is observed; the best fit values of $\mu _\text {SE}$ have ∼5% standard deviation with average error bars of ∼6%, and exhibit no obvious trends.

A potential explanation for multiple electron emission is that 128 nm electroluminescence photons may produce secondary electrons by photo-ionizing the stainless steel extraction grid. The photoelectric yield of stainless steel is measured to be 0.8% at 130 nm and 1.2% at 124 nm in Ref. [50]. The grid’s transparency at normal incidence is 95%, and the electroluminescence yield ranges from 120 photon/e$^{-}$ beneath the central PMT to 80 photon/e$^{-}$ beneath the inner ring of PMTs [31]. Assuming that half of these UV photons are directed towards the grid, these values predict a 2–4% probability of a 1 $\text {e}^{-}$ event ionizing the grid. This is consistent with the best-fit value of $\mu _\text {SE}$.

Secondary electrons reach the gas pocket ∼ 1.4 μs after the photon from the first electron strikes the extraction grid, since the LAr layer extends 4.7mm above it, and electrons travel with 3.1–3.4 mm/*rmu*s drift speeds in the 2.8–3.7 KV/cm extraction field [51]. This time difference is small compared to the 3.4 μs slow component lifetime of electroluminescence [2], so the pulse-finding algorithm fails to identify them as a separate S2 pulse.

**Fig. 19:**
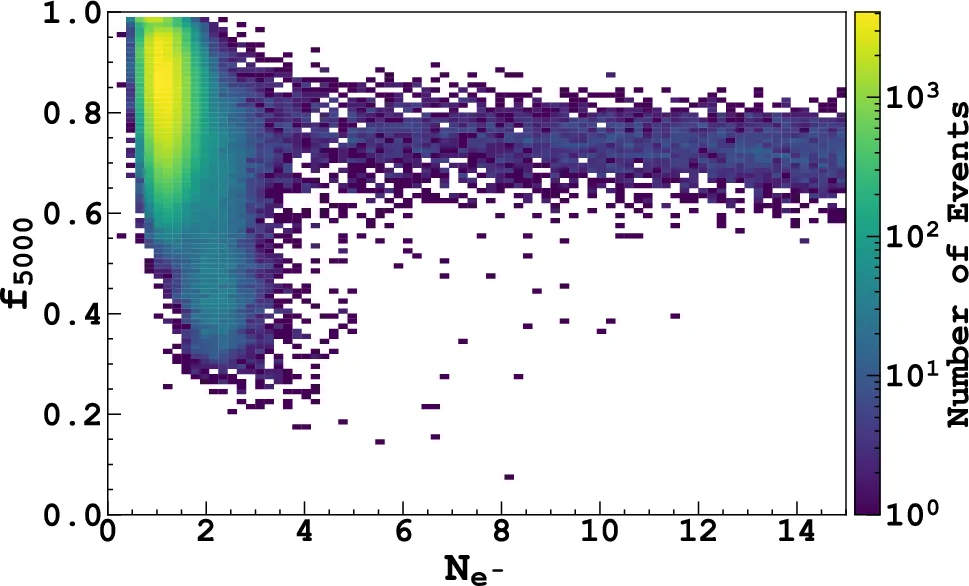
Pulse shape parameter, $f_{5000}$, vs. $N_{e^-}$. The lower $f_{5000}$ of the distribution around $N_{e^-}$ = 2–3 indicates more spread pulses than $N_{e^-}>4$. The z-axis is the number of events in each bin

Evidence of secondary electrons delayed by ${\mathcal {O}}({1\,\mathrm{\upmu {s}}})$ can be also be seen in SE pulse shapes. In Fig. 19, a pulse shape parameter, $f_{5000}$, the fraction of light in the first 5 μs of the entire pulse, is shown as a function of $N_{e^-}$. The population between $N_{e^-}= 2$ and $N_{e^-}= 3$ extends to lower $f_{5000}$ than the population with $N_{e^-}>4$, showing clear distinction in the pulse shape. The lower mean $f_{5000}$ value for two to three electrons indicates that electrons reach the gas pocket at different times while the pulse finder reconstructs them as a single pulse. This behavior is seen qualitatively in recorded waveforms: two separate pulses separated by ${\mathcal {O}}({1}{\upmu {s}})$ are observed for SEs with $N_{e^-}>1$. Further investigations of the pulse finder algorithm’s behavior at low PE are needed to fully test this hypothesis, and is left to future work.

## Discussion

While there may be multiple SE sources, these observations suggest two phenomenological classes: *temporally-correlated* SEs and *uncorrelated* SEs.

### Temporally-correlated SEs

The temporally-correlated SEs appear to have a parent event with which they demonstrate strong spatial and temporal correlation (separated by an exponential time constant on a 1–1000 ms time scale), and the probability of observing a correlated SE scales linearly with the parent’s S2 size and $t_\textrm{drift}$. There are at least three components: a fast component of ∼5 ms ($\tau _1$), a slower component of ∼40–80 ms ($\tau _2$), and a component of ∼16 ms that appears when the getter is bypassed from the circulation loop. Figure 16 shows that $R_2$ fluctuations correlate with radon trap temperature. Those observations suggest that the cause of the correlated SEs is impurities in the LAr volume, and there are at least three species causing $\tau _1$, $\tau _2$, and the component with the getter off. There may also be an SE population correlated with parent events by a long enough delay that parent-SE pairs are not correctly identified.

The following are observations that hint at species of the impurities. (1) The stable SE component with $\tau _1$ may exist primarily in the liquid and be unaffected by the gas-phase recirculation and purification system. It may not be removed by the getter or at an equilibrium concentration, given some constant source. (2) If the population described by $R_2$ in Fig. 7 results from two impurities, the change of $\tau _2$ after 200 days may be explained by the presence of a volatile impurity with $\tau _2{\sim 80\,\mathrm{\text {m}\text {s}}}$ that is removed, leaving a less volatile one with $\tau _2{~40}{ms}$. (3) The correlation with radon trap temperature may indicate that at least one impurity enters the gas phase and is partially removed by the cold trap, since the adsorption of charcoal in the radon trap increases when it is colder. (4) The new component that appeared when the getter is bypassed from the circulation loop may be explained by the introduction of an impurity that is volatile at 87 K and mainly stays in the gas phase since this component was removed within a short time (36 h) by the recirculation system. (5) Based on the fact that in the getter-off runs, there is no noticeable degradation in the electron lifetime, at least the impurity causing SE events with the time constant of ∼16 ms in the getter-off data must be different from the impurity causing the electron lifetime loss.

Although the high electronegativity of ${\textrm{O}_{2}}$ makes it a candidate explanation for $\tau _e$, it may also prevent ${\textrm{O}_{2}}$ from releasing electrons as SEs. ${\textrm{N}_{2}}$ may be a good candidate consistent with some of the above observations, as it boils at 77 K and captures electrons in LAr with an attachment coefficient ∼1% that of ${\textrm{O}_{2}}$ [52]. Also, ${\textrm{N}_{2}}$ has slightly higher electron affinity of −0.07 eV than Ar (−0.1 eV) and in Ar media, the effective electron affinity of ${\textrm{N}_{2}}$ could be positive. However, the triplet lifetime study suggests that the ${\textrm{N}_{2}}$ contamination was <1 ppm level as shown in Sect. 4.2. The long lifetime of metastable anions that produce SEs, according to this mechanism, can potentially be explained by long resonant autodetachment times typically seen in diatomic molecular anions, from long-lived vibrational states (prolonged at low temperatures) [53] or small energy level gaps [54]. Similar mechanisms make ${\textrm{OH}^{-}}$ or residual moisture plausible SE sources from non-volatile impurities. TPB is another candidate: it is soluble in LAr [55] and scintillates with ${\mathcal {O}}({1\,\mathrm{\text {m}\text {s}}})$ lifetimes [56] (implying the existence of long-lived molecular excited states consistent with lower rates of inter-system crossing in TPB’s molecular structure), and similar benzenoid molecules have long-lived anionic states [57, 58].

The hypothesis that impurities cause the temporally-correlated SEs is supported by the observation. However, to confirm the hypothesis, identify impurities, and develop means to reduce SE events in future experiments, the SE rate needs to be studied with spiked impurity concentrations in a dedicated system.

### Uncorrelated SEs

For uncorrelated SEs, no parent has been identified within the ∼1 s timescales accessible in this study. This component is either from spontaneous causes or correlated SE components on a time scale >1 s. Figure 9 shows that a component extends out to ≈10s timescales at a rate comparable to $R_1$ and $R_2$. Due to its long lifetime, sufficient statistics are only achieved with a long period, making it hard to study this component in the same way as the other shorter components. It is possible that all or most of the so-called uncorrelated SEs actually do have a parent event that has not been identified. This explanation is supported by the observation that the uncorrelated SE rate in Fig. 10 is proportional to the rate of ionization. One of the possible mechanisms for this long-time correlation is that negative ions drift to the extraction region and release electrons. Some parents may also be missed by the selection criteria, though this is unlikely to be a dominant source of uncorrelated SEs given the high acceptance of these criteria and the low SE production rate for the low-energy events that fail cuts, as shown in Fig. 12.

The upper bound on the constant component of the best-fit line to the “uncorrelated” SE rate in Fig. 10 implies that any SE production mechanisms truly unrelated to ionization of the LAr must have a rate <7.5 mHz. Such mechanisms can include spontaneous electron emission from the grid [59, 60]. While spontaneous emission of electrons trapped at the gas/liquid interface has been proposed as an SE-production mechanism in LXe experiments [23, 24, 41], this mechanism is unlikely to explain SEs in DarkSide-50, as the electron-transparency of the liquid surface is much higher in LAr than LXe [61], and the corresponding fast component of trapped-electron emission is not observed [62].

### Comparisons with Xenon experiments

Dual-phase liquid xenon experiments have also observed SE signals. A study of SEs in the XENON1T experiment concluded that they were both temporally and spatially correlated with preceding high-energy events [42]. The time-correlated SEs in XENON1T follow a power law. On the other hand, in DarkSide-50  at least one component of SEs released following a parent event shows a clear exponential dependence, described by $\tau _2$. This suggests that at least one different mechanism for SEs exists in LAr compared to LXe. Power-law exponents differed between 1 $\text {e}^{-}$ SEs and multi-electron SEs, indicating that those with $N_{e^-}> 1$ were not simply the result of pile-up, but were rather produced by some mechanism capable of trapping and releasing more than one delayed electron.

A study of SEs in the LUX detector [41] found that SEs were primarily caused by two populations: bursts of electrons trapped under the liquid surface, and drifting electrons trapped by impurities. While evidence for the former mechanism is not observed in DarkSide-50, delayed release from impurities is identified as a potential source of correlated SEs in this work. Similarly to the XENON1T experiment, the delay times between SEs and their parents in LUX followed a power-law.

Extended electron tails on time scales shorter than the drift length have not been observed immediately following S2 pulses in DarkSide-50; the S2 pulse shape is consistent with the expected electroluminescence pulse shape [2] and shows no evidence of electrons delayed at the liquid/gas interface, contrary to what has been reported in xenon TPCs [23, 24, 41].

## Summary

Events with fewer than four extracted electrons behave differently from other S2-only events. Some fraction of these SE events ($\sim 30{-}70$% of SEs, Fig. 8) are likely electrons released from impurities. These events are correlated to preceding events in time, position, and energy. The rate of this correlated component increases with the parents’ S2 size and drift time. From these correlations, a trapping probability of $1.74\times 10^{-8}~\text {mm}^{-1}$ drift length is estimated with the getter on. A study of temporal correlations suggests that the timescale at which these trapped electrons are released from impurities is dominated by an exponential distribution with at least two components: a fast component of ∼5 ms ($\tau _1$), a slower component of ∼40–80 ms ($\tau _2$), and a component of ∼16 ms that appears when the getter is turned off. The change in the value of $\tau _2$ over time may indicate that this time constant describes two different components with changing relative abundances. We also note the existence of a longer-lived (>1 s) component not described by this exponential model. Observations are consistent with the hypothesis that uncorrelated SE events are correlated SE components with a lifetime of >1 s.

The electron multiplicity in SE events is well described by a Poisson distribution characterized by a mean number of electrons $\mu _\text {SE}$. The $\mu _\text {SE}$ does not depend on the impurity species, parent’s drift time, and parent’s S2 size. The $f_{5000}$ parameter in Fig. 19 indicates that the electrons in the SEs events with 2- and 3-electron multiplicity are not extracted into the gas pocket simultaneously. Those observations are consistent with the hypothesis that electroluminescence photons ionize the extraction grid, resulting in secondary electrons. If the photo-ionization yield of an extraction grid could be suppressed, the analysis threshold, which is limited by the leakage of SE events currently, might be lowered in future experiments. Confirmation of this mechanism will allow SEs with $N_{e^-}>1$ to be properly included in background models, allowing searches for low-mass DM at even lower thresholds than current generation experiments, and thus to probe lower masses and cross sections.

## Data Availability

This data will be made available on reasonable request. [Authors’ comment: The datasets generated during and/or analysed during the current study are available from the DarkSide-50 Collaboration on reasonable request].

## Code Availability Statement

This code/software will be made available on reasonable request. [Authors’ comment: The code/software generated during and/or analysed during the current study are available from the DarkSide-50 Collaboration on reasonable request].
